# Genomic Environment Predicts Expression Patterns on the Human Inactive X Chromosome

**DOI:** 10.1371/journal.pgen.0020151

**Published:** 2006-09-29

**Authors:** Laura Carrel, Chungoo Park, Svitlana Tyekucheva, John Dunn, Francesca Chiaromonte, Kateryna D Makova

**Affiliations:** 1 Department of Biochemistry and Molecular Biology, Pennsylvania State University College of Medicine, Hershey, Pennsylvania, United States of America; 2 Center for Comparative Genomics and Bioinformatics, Pennsylvania State University, University Park, Pennsylvania, United States of America; 3 Department of Biology, Pennsylvania State University, University Park, Pennsylvania, United States of America; 4 Department of Statistics, Pennsylvania State University, University Park, Pennsylvania, United States of America; 5 Department of Pediatrics, Case Western Reserve University School of Medicine, Cleveland, Ohio, United States of America; 6 Department of Health Evaluation Sciences, Pennsylvania State University College of Medicine, Hershey, Pennsylvania, United States of America; Fred Hutchinson Cancer Research Center, United States of America

## Abstract

What genomic landmarks render most genes silent while leaving others expressed on the inactive X chromosome in mammalian females? To date, signals determining expression status of genes on the inactive X remain enigmatic despite the availability of complete genomic sequences. Long interspersed repeats (L1s), particularly abundant on the X, are hypothesized to spread the inactivation signal and are enriched in the vicinity of inactive genes. However, both L1s and inactive genes are also more prevalent in ancient evolutionary strata. Did L1s accumulate there because of their role in inactivation or simply because they spent more time on the rarely recombining X? Here we utilize an experimentally derived inactivation profile of the entire human X chromosome to uncover sequences important for its inactivation, and to predict expression status of individual genes. Focusing on Xp22, where both inactive and active genes reside within evolutionarily young strata, we compare neighborhoods of genes with different inactivation states to identify enriched oligomers. Occurrences of such oligomers are then used as features to train a linear discriminant analysis classifier. Remarkably, expression status is correctly predicted for 84% and 91% of active and inactive genes, respectively, on the entire X, suggesting that oligomers enriched in Xp22 capture most of the genomic signal determining inactivation. To our surprise, the majority of oligomers associated with inactivated genes fall within L1 elements, even though L1 frequency in Xp22 is low. Moreover, these oligomers are enriched in parts of L1 sequences that are usually underrepresented in the genome. Thus, our results strongly support the role of L1s in X inactivation, yet indicate that a chromatin microenvironment composed of multiple genomic sequence elements determines expression status of X chromosome genes.

## Synopsis

To match the amount of gene product produced in males (XY), most genes in mammalian females (XX) are active on one X chromosome and inactivated on the other. However, some genes “escape” inactivation and are expressed from both X chromosomes. This study investigates sequences that may control whether a gene undergoes or escapes X chromosome inactivation, including DNA sequences previously thought of as non-functional or “junk.” Earlier work suggested that one such sequence, L1 interspersed repeats, may be associated with inactivation, but the extent of such association, and whether it represented a consequence of the evolutionary history of X, remained unclear. This study utilized recently generated chromosome-wide data on sequence and gene expression for human X, with a particular focus on the Xp22 region, which is evolutionarily young and has had no time to accumulate many L1 elements. A rigorous statistical analysis identified with high accuracy a set of short sequences that discriminate between genes undergoing and those escaping X chromosome inactivation. Interestingly, the majority of such sequences enriched in the vicinity of inactivated genes were found within L1s. These results strengthen the case for an involvement of L1s in X chromosome inactivation and suggest other DNA elements that might also play a role.

## Introduction

X chromosome inactivation (XCI) is an extraordinary example of long-range gene regulation, extending over 150 Mb (megabases) and transcriptionally silencing genes on one X chromosome in females in order to equalize X-linked gene dosage with XY males (reviewed in [1,2]). XCI initiates during early embryogenesis and requires the presence of the *XIST* gene (in *cis*), whose RNA transcript closely associates with and coats the inactive X chromosome [2]. Upon inactivation, the X chromosome is heavily epigenetically modified in many ways typical of other silenced loci, including the incorporation of methylated DNA and modified histones [3].

Notwithstanding the chromosome-wide nature of XCI, not all genes on the X are silenced [4–6]. These genes that “escape” XCI lack at least some epigenetic alterations characterizing the rest of the chromosome [5]. Recently, in conjunction with completion of the sequence of the human X chromosome [7], a comprehensive human X inactivation profile was established [6]. A total of 15% of assayed genes escape XCI; their distribution and organization is highly non-random and mirrors the evolutionary history of the X. The X-specific portion of the X is partitioned into five strata that show increasing levels of sequence divergence with increasing distance from the distal tip of Xp [7,8]. Genes that escape inactivation are primarily found within the youngest strata that map to Xp22. Furthermore, such genes are clustered, suggesting that they are controlled at the level of chromosome domains [6,9].

Consideration of escape genes is important for understanding how XCI spreads and is maintained in *cis* along the chromosome. Specific *cis*-acting sequences on the X may direct chromatin modifications or *XIST* RNA to specific sites along the chromosome, or might be involved in other aspects of regulating XCI. Studies of X;autosome translocations in human and mouse, and analysis of ectopic X inactivation of mouse *Xist* transgenes lend support for the involvement of *cis* regulatory sequences in the spreading of XCI. Although autosomal sequences on these chromosomes can be inactivated, autosome gene inactivation and spreading of *XIST* RNA as well as of epigenetic markers of inactivation are incomplete and in some cases discontinuous [10–15]. These studies suggest that the X may be organized in a manner distinct from that of autosomes and may be more receptive to transcriptional inactivation.

Such observations led Gartler and Riggs to hypothesize that specific sequences, “booster elements” or “way stations,” could propagate an inactivation signal [16]. Such sequences need not be unique to the X, but should be more highly represented on the X than on autosomes. Subsequently, Lyon proposed that the repetitive element LINE-1 (L1) may function as such a booster [17], based on cytological studies showing L1 enrichment on the X in human and mouse [18,19]. Complete sequencing of the X confirmed this enrichment; L1 elements are approximately 2-fold enriched on the X compared to autosomes [7]. However, the distribution of L1 elements fluctuates along the X, with the highest proportion in the evolutionarily oldest strata. Notably, preliminary analysis suggested that sequences adjacent to genes escaping inactivation are depleted in L1s [20], although this study did not consider differences in L1 density along the X chromosome and escape gene organization. Moreover, although the study by Bailey et al. [20] lent support to the L1 hypothesis, it did not consider an alternative (but not mutually exclusive) model: escape genes may be associated with different *cis* regulatory sequences that prevent these genes from either initiating or stably maintaining inactivation.

The recently established X inactivation profile and completed X chromosome sequence [6,7] prompted us to reinvestigate the role of genomic sequences in XCI. To find sequences that may influence X inactivation state, we computationally identified overrepresented motifs in the neighborhoods of both inactivated genes and genes that escape XCI in Xp22. These enriched sequences correctly predict the inactivation state of most genes along the entire X chromosome.

## Results

### Description of the Escape and Inactivated Subgenomes Analyzed in Xp22

We focused our analysis on the Xp22 region for the following reasons. First, Xp22 contains about equal numbers of genes that are transcriptionally silent on inactive X and of genes that escape inactivation. In fact, among 103 genes assayed in Xp22 [6], 30% (31 genes) are subject to inactivation and 39% (40 genes) escape inactivation. (The other genes exhibit heterogeneous expression patterns between different inactive Xs tested.) This is in contrast with the rest of the X chromosome, where the overwhelming majority of genes are inactivated (66%, or 339 out of 515 genes assayed), and only a small percentage of genes escape inactivation (6%, or 31 genes) [6]. Second, within Xp22, inactivated genes and genes escaping XCI are located in the same, relatively young, evolutionary strata (part of stratum 3 and strata 4–5). Thus, comparison of silenced and escape genes within Xp22 is expected to highlight XCI signals and not the evolutionary differences between strata. Third, we hypothesized that, if L1 interspersed repetitive elements were involved in XCI, analysis of a region in which their overall density is low could reveal either local L1 organizational differences or additional XCI regulatory elements. Indeed, only 15% of the Xp22 sequence is covered by L1 elements, as compared with 29% for the whole X chromosome [7]. The pseudoautosomal region (also located in Xp22) was excluded from our analysis.

To delineate sequences determining the inactivation status of genes in Xp22, we divided this region into two subgenomes, *I* (for inactivated) and *E* (for escaping inactivation). The Xp22 genomic sequences were compiled on the basis of the X inactivation profile [6], including regions upstream and downstream from the transcription start site (TSS) of each gene. We considered three distances surrounding the TSSs of genes: ±50 kilobases (kb), ±100 kb, and ±250 kb. Thus, based on these distances, three pairs of *I* and *E* subgenomes were investigated: *I*_50_ and *E*_50_, *I*_100_ and *E*_100_, and *I*_250_ and *E*_250_ (Table 1). Each subgenome consisted of several “contigs”: uninterrupted genomic sequences upstream and downstream of the TSS of a gene with a particular expression pattern. Frequently, the region surrounding a specific gene overlapped the region surrounding an adjacent gene with the same expression profile. In this case, the genomic sequences around TSSs of both (or sometimes several) genes were merged into the same contig. Overlapping surrounding sequences of adjacent genes with *different* inactivation patterns were excluded. The subgenome pairs were constructed to keep the frequency of repetitive elements and genomic length approximately equal between the two subgenomes (Table S1). Notably, the frequency of only one type of repetitive element (ERV class I) differed by more than 2-fold between any two subgenome pairs. L1 repeats were at low frequency in both subgenomes, but were slightly more abundant in the *I* subgenomes compared to the *E* subgenomes (1.4- to 1.6-fold difference), e.g., the L1 difference in the ±50-kb subgenomes is 13.3% versus 9.7%, both notably lower than the 29% X chromosome average [7].

**Table 1:**
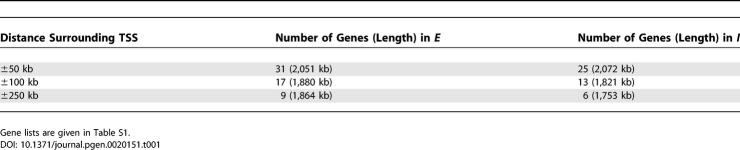
Gene Number and Length of Contigs for the *E* and *I* Subgenomes within Xp22 (Used to Discover Overrepresented Oligomers)

### Analysis of Oligomers Enriched in Either *E* or *I* Subgenomes

We next developed an XCI profile–driven computational approach to contrast genomic sequences adjacent to genes that are inactivated or escape inactivation. We compared the frequency of all possible oligomers of specified length between the *I* and *E* subgenomes. Initially, 8-, 12-, 16-, 20-, and 24-mers were examined separately for each of the three subgenome pairs. An oligomer was considered to be overrepresented in a subgenome if (1) it was present at least ten times in that subgenome; and (2) its frequency was at least 5-fold higher in that subgenome compared to the other subgenome. The oligomers that were identified using these initial criteria were further evaluated with a permutation test (see Methods) that assessed statistical significance of the overrepresentation. We focused our further analysis on 12-mers because they had the highest total number of different oligomers overrepresented for the *E* or *I* subgenomes.

**Figure 1:**
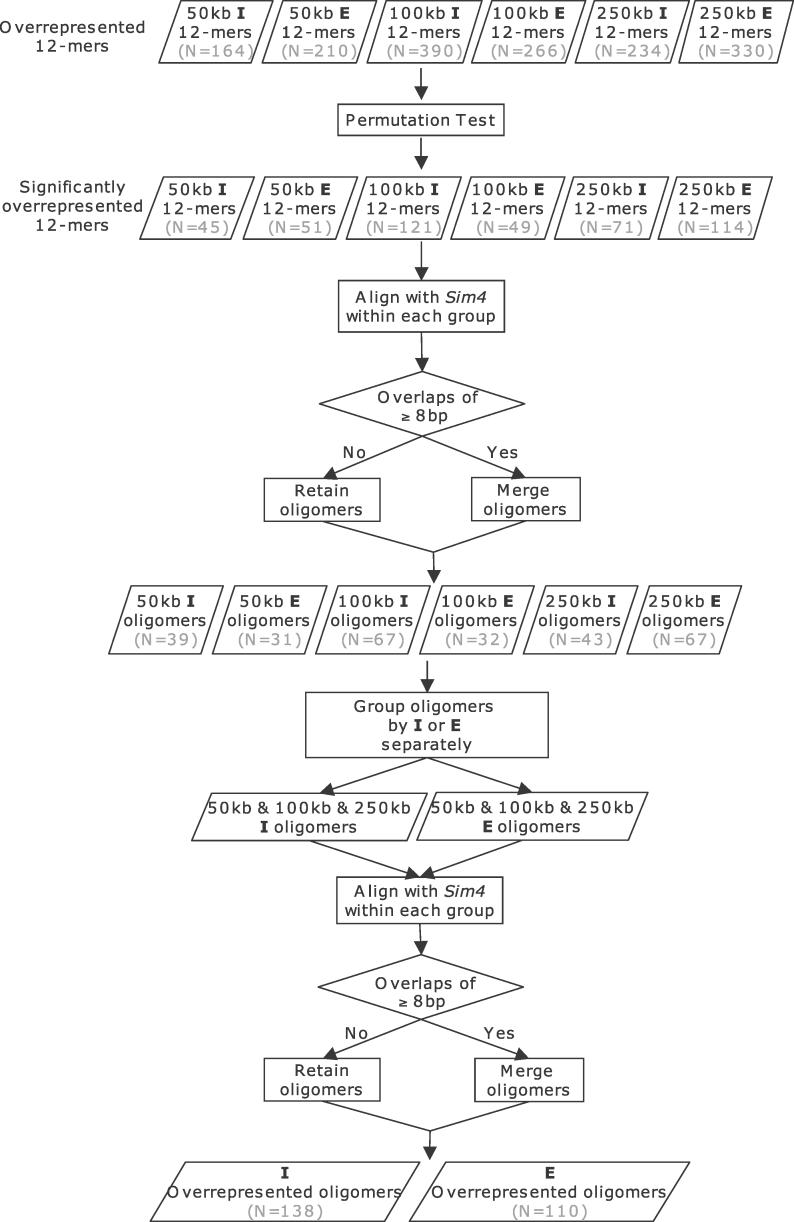
Procedure Used to Obtain Overrepresented Oligomers Starting from the Overrepresented 12-Mers The overrepresented 12-mers were defined with the initial criteria: at least ten occurrences and at least 5-fold enrichment. See Results and Methods for a detailed description.

Two additional operations were performed on the significantly (*p* < 0.01) overrepresented 12-mers (Figure 1). First, overlapping 12-mers were merged into longer oligomers. Second, such oligomers identified at different distances surrounding genes (±50 kb, ±100 kb, or ±250 kb) of the *E* subgenome were pooled (and merged) into a single set. This allowed the oligomers that were found to be overrepresented only at one or two distances to be considered in the further analysis of all three distances from TSSs. We followed the same procedure for 12-mers identified in the *I* subgenome. The resulting set consisted of 110 and 138 different oligomers overrepresented for the *E* and *I* subgenomes, respectively (Figure 1, Table S2). These are called “overrepresented oligomers” in the remainder of the manuscript. Remarkably, the majority of overrepresented oligomers (74% for *E* and 60% for *I*) were also significantly enriched on the X chromosome compared with autosomes (*p* < 0.05, permutation test). Focusing only on the oligomers enriched on chromosome X compared to autosomes had little effect on our quantitative results and did not alter our conclusions (unpublished data); therefore, all 248 (110 + 138) overrepresented oligomers were used in the analyses described below.

**Figure 2:**
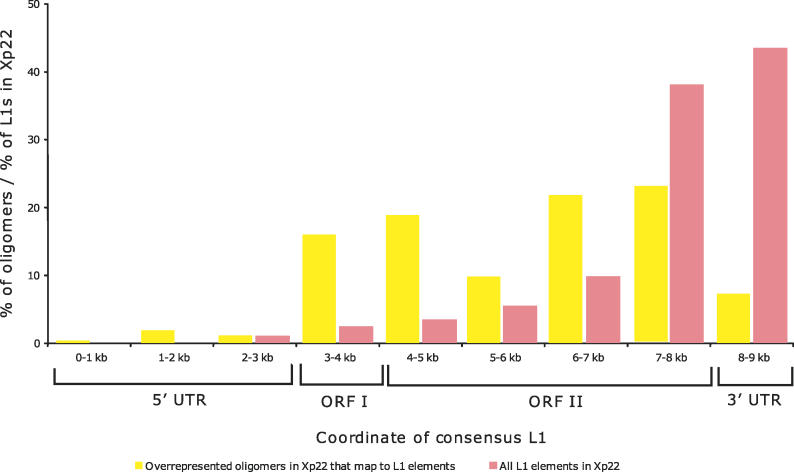
The Distributions over the Length of L1 Element of Overrepresented Oligomers Found in *I* Subgenome (Yellow Bars) and of All L1 Sequences within Xp22 (Red Bars) Only overrepresented oligomers mapping frequently to L1s (>50% of their genomic occurrences in the *I* subgenome) are shown. Although the full-length L1 is approximately 7 kb long, the alignment of L1 subfamilies was approximately 9 kb long. ORF, open reading frame.

Interestingly, oligomers overrepresented in the *E* and *I* subgenomes mapped within different sequence classes (Table 2). Indeed, 38% of oligomers overrepresented in the *E* subgenome were located within *Alu* repeats (the corresponding value for the *I* subgenome is only 9%). In contrast, 64% of the oligomers overrepresented in the *I* subgenome were within L1 repeats (compared with only 4% for *E*). Intriguingly, although the majority of L1 sequences in Xp22 (as well as on the X chromosome and in the whole human genome) are truncated at the 5′ end and frequently include only 3′UTR sequences [21], the oligomers enriched in the *I* subgenome had a substantially different distribution; they were enriched in ORF1 and ORF2, but depleted from the 3′UTR (Figure 2).

**Table 2:**
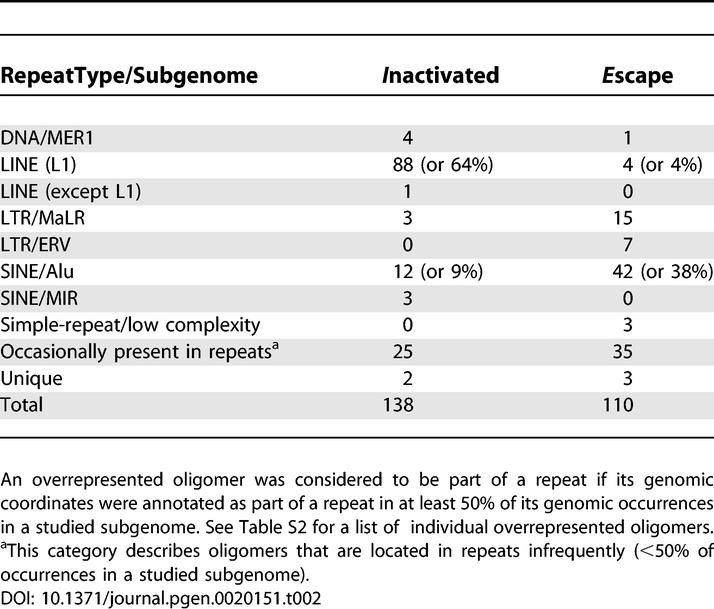
Assignment of Overrepresented Oligomers to Interspersed Repetitive Elements (Repeats)

### Classification of Genes as Either Inactivated or Escaping Inactivation Based on Surrounding Oligomers

To predict the inactivation status of genes (either *E* or *I*), we used linear discriminant analysis (LDA) [22]. In the application of LDA to the present study, genes were units to be classified, and counts of overrepresented oligomers surrounding TSSs of genes were classification features. Our training data consisted of the Xp22 genes that comprised our original *E* and *I* subgenomes, together with additional X chromosome genes for which expression status had been confirmed by a second assay in primary fibroblasts [6] (Tables 3 and S3). Although overrepresented oligomers were derived from Xp22, extending the training set with additional X chromosome genes allowed us to “learn” the role of these sequence elements in XCI, not just within Xp22, but more generally on X.

**Table 3:**
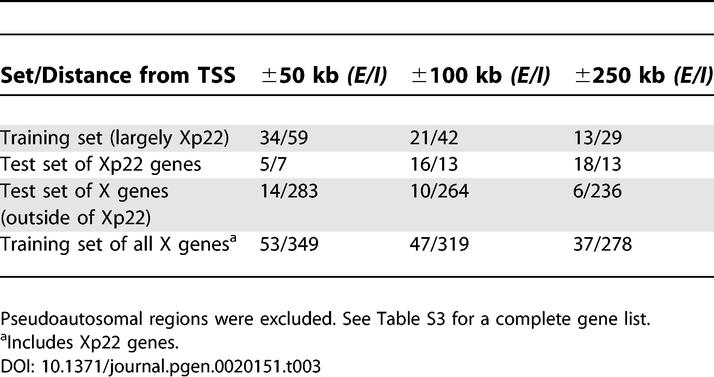
The Numbers of Genes Analyzed for Training and Test Datasets

**Figure 3:**
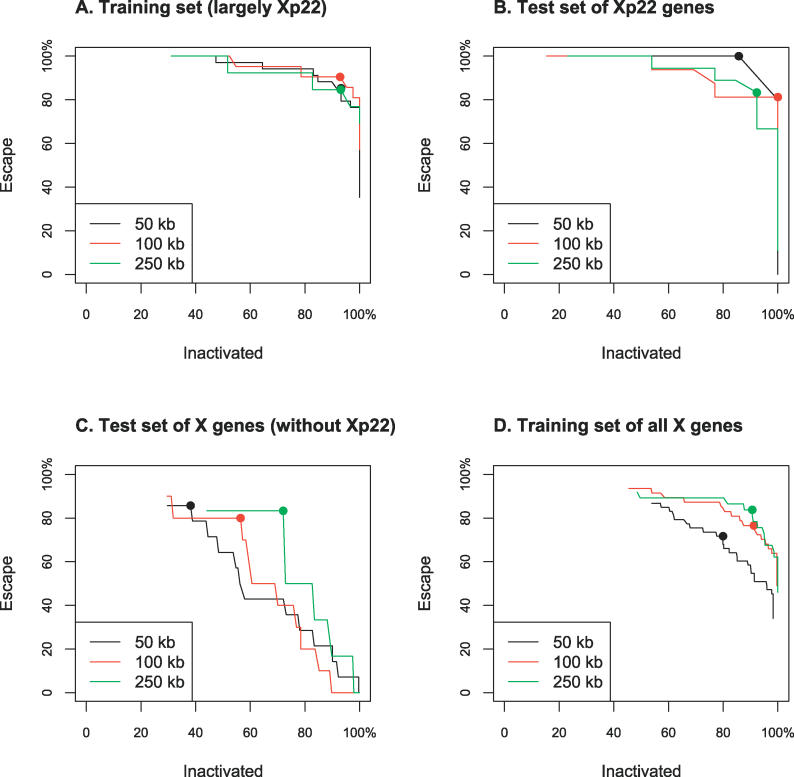
LDA Classification Success Rates for Different Values of the Tuning Parameter τ (A) Training set derived largely, but not exclusively, from Xp22 (See Table S3). (B) Test set of Xp22 genes, with training performed on genes in (A). (C) Test set of X genes outside of Xp22, with training performed on genes in (A). (D) Training set of all X genes, including genes in Xp22. Dots indicate optimal values of τ (see Table 4 and Methods).

LDA based on counts of overrepresented oligomers had excellent performance on the training set; correct classification rates assessed by leave-one-out cross-validation were ≥85% for both *E* and *I* classes at each of the three distances surrounding TSSs (Figure 3A, Table 4). Next, we investigated whether the LDA classifier trained this way could predict expression status in two non-overlapping test sets, namely: (1) Xp22 genes not used in training, and (2) X chromosome genes outside of Xp22 and excluding pseudoautosomal regions; here also, training set genes were not included (Table 3 and S3). For these two sets, the counts of the overrepresented *E* and *I* oligomers (originally discovered from Xp22) surrounding each gene were calculated, and the XCI state was predicted.

**Table 4:**
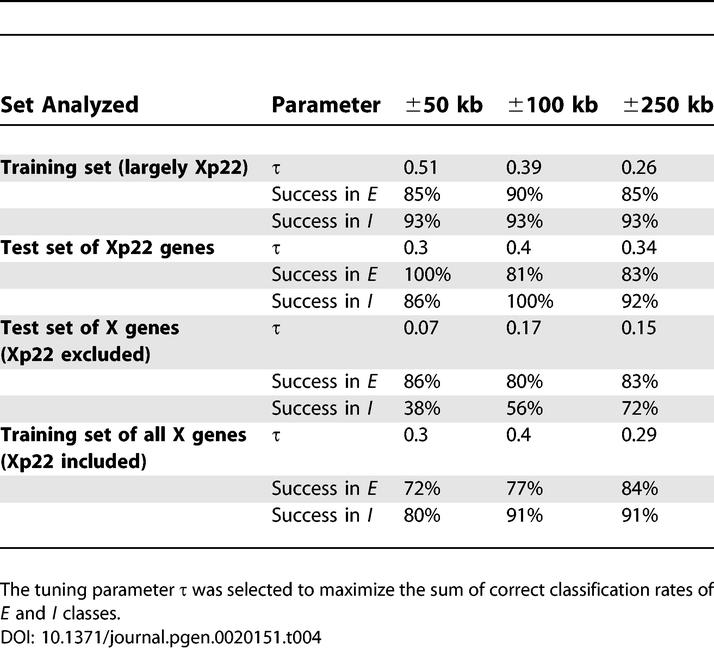
Success Rates of LDA

We achieved high correct classification rates (≥81%) for Xp22 test genes at all three distances examined (Figure 3B, Table 4). Thus, our classifier effectively captures crucial genomic differences between *E* and *I* genes in the Xp22 region. Classification performance for X chromosome test genes increased with the distance surrounding TSSs (Figure 3C, Table 4). At ±250 kb, we were able to reach correct classification rates of 83% and 72% for *E* and *I* genes, respectively, whereas performance at ±50 kb and ±100 kb was somewhat lower. Higher performance on Xp22 than on other X chromosome test genes could be due to the fact that the training data largely included Xp22 genes. As a consequence, the classifier may be capturing genomic features prevalent in Xp22 in addition to the XCI signals we are seeking.

To overcome this problem, we used overrepresented oligomers derived from Xp22 to train LDA on all X chromosome genes (excluding pseudoautosomal regions). In other words, we replaced our original, mostly Xp22-based training set with a new one comprising genes from the entire X chromosome. The new set included all genes from the initial training set as well as from the two test sets discussed above. Leave-one-out cross-validation on this new, chromosome-wide training set yielded correct classification rates of 84% and 91% for *E* and *I* genes, respectively, for ±250 kb from TSS (Figure 3D, Table 4). This represents a substantial improvement in performance relative to previous success rates for the test set of X chromosome genes (see above). Moreover, the chromosome-wide training may in fact be less influenced by Xp22 “landscape” features and thus captures XCI signals more effectively. Subsequent results concerning correctly and erroneously classified genes were based on the outcomes of this analysis.

### Genes Classified Correctly and Misclassified Genes

**Figure 4:**
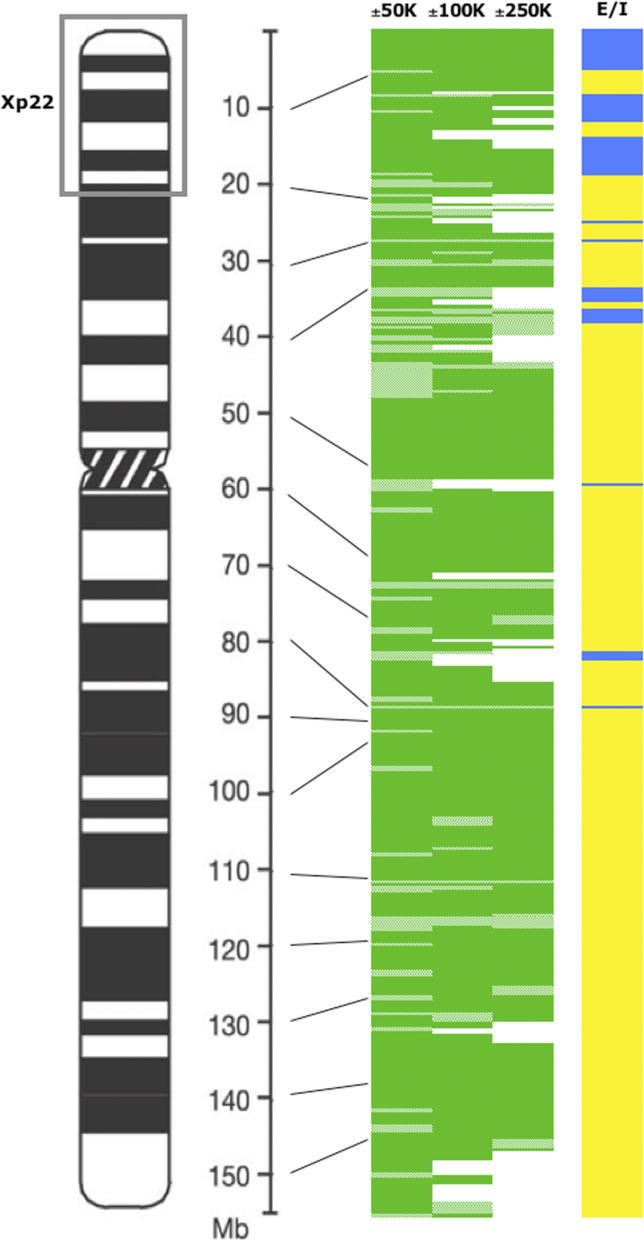
The Distribution of Correctly and Incorrectly Classified Genes along the X Chromosome Dark green indicates correctly classified genes; light green indicates misclassified genes. X inactivation expression patterns [6] for genes included in this study: yellow indicates inactivated genes, and blue indicates escape genes. Not all genes were analyzed at all distances because sequences that included adjacent genes with *different* inactivation patterns were excluded from analysis (see Methods). These gene distances remain uncolored.

Classification performance for LDA trained on all X chromosome genes is visualized with respect to chromosome location in Figure 4. Large *E* and *I* domains are, for the most part, well predicted. Our overall high rate of correct classification, despite evolutionary differences that have influenced the X sequence composition, argues strongly that these enriched oligomers successfully capture differences in XCI and not simply genomic differences in X chromosome sequence.

Notwithstanding the good overall classification performance, chromosome location does seem to influence our ability to predict inactivation status, as specific regions have higher assignment errors for both *E* and *I* genes. This is most apparent for genes within Xp11.3–Xp11.4 (~40–48 Mb). The reasons for incorrect classifications in this particular region are puzzling. Gene density and repeat content fluctuate greatly within Xp11.3–Xp11.4; however, genes at other X chromosome locations with even more dramatic fluctuations in these parameters are classified correctly. The region also contains an evolutionary breakpoint between strata 2 and 3, although it is unclear what role this could play in misclassification of both *E* and *I* genes.

Escape genes are particularly well classified within Xp22 domains. A plausible explanation is that the overrepresented oligomers were derived from within this region, although from a small subset of the correctly classified genes. Nonetheless, very large escape domains are not present elsewhere on the X, and smaller escape regions may not show adequate enrichment for classification purposes. Supporting this idea, we failed to correctly classify the only two non-domain escape transcripts, Hs.458197 and *SH3BGRL,* included in this study (at all three distances from TSSs); other non-domain escape genes were omitted because of the proximity of adjacent inactivated genes. In another instance, both *E* and *I* transcripts in and surrounding a <250-kb escape domain in Xp11.1 (including KIAA0522) are assigned incorrectly at the only scorable distance, ±50 kb. This could suggest that both *E* and *I* signatures were detected, but that classifications were confounded by nearby genes of differing inactivation status. Although chromosome-wide classifications were most successful at ±250 kb from the TSS, domains of a different size may have different signatures, and analysis of smaller distances may be necessary to correctly assess a larger number of escape genes outside of Xp22. Classification performance on the whole X also likely reflects repeat element landscape differences for both *E* and *I* genes. At ±250 kb, misclassified *I* genes have strikingly lower L1 concentration than correctly classified genes (17.4%, *n* = 26 genes, versus 24.3%, *n* = 252 genes), whereas L1 concentration of misclassified *E* genes is much higher than at their correctly assigned counterparts (27.6%, *n* = 6 genes, versus 11.1%, *n* = 31 genes).

## Discussion

Unlike elsewhere on the X, an extraordinarily high proportion of genes in Xp22 escape X inactivation [6]. For this reason, and because these sequences have similar evolutionary origin, we rooted our computational approach within Xp22 in an effort to identify regulatory elements involved in XCI. This reduced the risk of uncovering genomic and evolutionary X chromosome features unrelated to XCI. Notably, using only oligomers identified as enriched in Xp22, we were able to successfully predict XCI status for the vast majority of genes along the entire X chromosome. The approach presented here is completely dependent on an experimentally derived XCI profile that was obtained by assaying human inactive X chromosomes in somatic cell hybrids [6]. A subset of these genes were validated in primary cell lines and confirmed that incorrect assignment of XCI status in the hybrids is rare [6]. Nonetheless, such genes would contribute to misclassification in the present study.

The hypotheses that interspersed regulatory elements (booster elements [16]) control spreading of XCI, and that other elements regulate escape genes, predict that the intrinsic sequence composition of regions surrounding genes with different inactivation statuses should differ. Our strategy made no assumptions about the identity of any overrepresented sequences; however, employing stringent enrichment criteria, we indeed found most overrepresented oligomers in both the *I* and *E* subgenomes within known classes of interspersed repeats. The potential regulatory role of repetitive elements is an emerging theme in epigenetics; other computational studies have reported increased density of interspersed repeats near imprinted loci that were utilized to predict similarly regulated genes [23,24]. The roles of repeats in both spreading and in escape from XCI are discussed separately below.

A high proportion of the enriched *I* oligomers map to L1 sequences. These results substantially strengthen the L1 hypothesis [17], as we started with a region of relatively low L1 frequency and were able to identify L1 sequences that are highly enriched near inactivated genes. The location of overrepresented oligomers within the L1 consensus sequence differs significantly from the L1 sequences within Xp22 or the whole X (Figure 2), suggesting that their enrichment may be functional and is not simply due to evolutionary mechanisms that have led to higher repetitive element levels on the X than on autosomes [7,25]. Nonetheless, the L1 oligomers found here predominantly map to primate-specific L1s (unpublished data), and presumably reflect the recent evolutionary origin of Xp22 [7,8] from which they were identified. Young L1s are attractive candidates for spreading XCI within Xp22 because it was hypothesized that formerly autosomal genes must acquire certain sequence characteristics to be inactivated [26]. A sequence necessary for spreading or maintaining XCI could function as a binding site for *XIST* RNA or heterochromatin proteins. Such a recognition motif would likely include non-conserved nucleotides and therefore be represented in older L1s as well. We are currently in the process of adapting our computational approach to score imperfect matches, which may effectively identify such sequences and improve classification success even further. Moreover, our study identified additional oligomers mapping to other repeats that were important for classification on the human X. The involvement of these sequences in XCI must be considered in future studies.

Are repetitive elements also involved in regulating escape from XCI? Previous X sequence analysis concluded that reduced density of L1s may be necessary but not sufficient to establish domains that escape XCI [20]. Our analysis has identified overrepresented oligomers in *E* subgenomes that may instead (or additionally) regulate expression of genes escaping XCI. The predictions were more accurate for escape genes in distal Xp than elsewhere on the chromosome (Figure 4). Perhaps this is because escape domains in this region are larger and, therefore, signatures are easier to capture. Alternatively, smaller escape domains or isolated escape genes may be regulated in a different manner and would explain why many were poorly classified. Sequences such as insulators, boundary elements, or barriers flank other coordinately regulated genes [27]. Functional analysis of the junctions between domains will be necessary to establish the role of these sequences or to identify other elements. Indeed, CTCF-bound insulators flank several escape genes [28], although their role in X inactivation has not yet been definitively established. Such sequences would not have been identified with the present approach, but may be further distilled from chromosome landscape features through a comparative study. On the human X, domains that escape inactivation largely include at least one gene with Y homology that would be required to escape inactivation for appropriate male:female gene dosage [6]. Repetitive element control of XCI could predict that boundaries of escape domains will shift in different species, but likely that the genes with Y homologs remain expressed on the inactive X. Notably, this prediction appears true for one domain studied in human and mouse [29]. Whether this prediction will hold elsewhere on the X and in different species is unknown because although X chromosome sequences are currently available for several mammals, a comprehensive XCI profile exists only for the human X.

Successful classification for most genes within large escape domains, particularly within Xp22, does support a role for enriched oligomers in regulating the expression of these genes. Many of these overrepresented oligomers map to *Alu* repeats. A plausible function for such escape motifs would be to prevent methylation at CpG islands. Interestingly, a previous study found that sequences within *Alu*s were good genome-wide predictors for CpG islands that were resistant to de novo methylation [30].

Another interesting oligomer overrepresented in the *E* subgenome maps to a simple repeat (GATA)_n_ that was recently proposed by others to determine escape from XCI [31]. We investigated this particular repeat, asking whether occurrences of (GATA)_n_ alone could predict the inactivation status of genes using a naive Bayes classifier (the same that was used with the LDA for oligomer-based classification). Training and test sets were defined and used as before. (GATA)_n_ performed well in predicting the status of the Xp22 genes used in training, yet was not as effective as our oligomer-based LDA predictions on the test sets. Indeed, even when cross-validating genes on the whole X chromosome, using (GATA)_n_ affords correct classification rates of at most 70% (Table S4). As (GATA)_n_ enrichment was identified by comparison of the most distal 7.5 Mb of Xp to the rest of the X chromosome [31], in contrast to the gene-directed approach that we have employed for our study, it is possible that overrepresentation of this simple repeat could reflect landscape differences unrelated to X inactivation or, in any event, could be only one of several discriminating factors.

Five oligomers did not initially appear to map within repetitive sequences. These oligomers were examined in more detail, and none is composed of a truly unique sequence. Two of the oligomers enriched in the *E* subgenome map to localized repeats within Xp22. The oligomer CAGTGGTTCTTCC is found within a 30-bp repeat motif within the VCX gene family that has multiple members mapping to Xp22.31. Similarly, AAAGCCAGTTAC is part of a tandem repeat that encompasses 650 bp, also within Xp22.31. It is not surprising that our enrichment strategy identified such repeated sequences, but their focused location makes them unlikely candidates to play a role in XCI. Three other oligomers, AAACCATATCAC, identified as enriched in *E* sequences, and *I-*enriched GGGCCGGGCGCA and AAAAATGTTTAA, were not found in repeats within the Xp22 subgenomes, according to our conservative definition requiring both start and end coordinates of an oligomer to be within the repeat. However, closer examination established that each of these frequently was directly adjacent to or occasionally overlapped with known repeat elements. Although, unfortunately, not identifying new candidates controlling X inactivation, these oligomers do give further support to the role of repeat sequences in predicting expression patterns on the chromosome.

Future efforts will focus on identifying motifs that may further improve prediction of XCI status. In this study we only considered inactivated and escape genes that were adjacent to genes with similar inactivation status. Further analyses will need to incorporate more complex patterns of inactive X expression, including genes within domains that show the opposite inactivation pattern, and heterogenous genes that escape inactivation only in a subset of inactive Xs tested [6]. Even for the genes considered in this study, it is very likely that additional parameters may provide substantial predictive contributions. Features to investigate include CpG islands, gene density, location within an escape domain particularly with respect to domain boundaries, and distance from the *XIST* locus. This idea is supported by a recent computational study that suggested L1 and *Alu* repetitive elements as important predictors for inactivated and escape genes respectively, and identified additional parameters that may also influence inactive X expression [32]. Genomic features that control XCI will further aid in our understanding of long-range control of gene expression and the impact of repetitive elements throughout the genome.

## Methods

### Transcripts.

We utilized a comprehensive inactivation profile of X chromosome genes assayed in fibroblast-derived somatic cell hybrids containing one inactivated X chromosome [6]. Genes were considered to be X inactivated if silenced in all nine somatic hybrids tested or if expressed in only a single hybrid (0/9 or 1/9). Genes were scored as escaping XCI if expressed in eight or nine out of nine somatic hybrids tested (8/9 or 9/9). The TSSs for X chromosome genes were from Supplementary Table S3 in [6]. We assumed positive strand to be the coding strand for genes represented by ESTs (expressed sequence tags) with unknown strand orientation. This assignment is not expected to influence our results because the majority of genes represented by ESTs with unknown strand orientation were shorter than 1 kb.

### Oligomer enrichment analysis.

A series of Perl programs (available upon request) were developed to analyze the genomic sequences located in the subgenomes. Each possible oligomer of a specified size (8-, 12-, 16-, 20-, and 24-mers) was sequentially counted within each subgenome. Exact matches were required. Counts of oligomers with reverse complementary sequence were combined.

To evaluate the significance of overrepresented 12-mers, we implemented a random permutation test for each of the three subgenome pairs (*E*_50_ and *I*_50_, *E*_100_ and *I*_100_, and *E*_250_ and *I*_250_) separately. Contigs were broken into nonoverlapping 2-kb fragments. *E* and *I* labels were removed, and the 2-kb fragments were randomly distributed to either a mock *I* or a mock *E* subgenome. The two mock subgenomes were equal in size. This process was repeated 1,000 times. To determine the empirical *p-*value for each 12-mer, we calculated the number of permutations in which this 12-mer was present at least ten times and overrepresented at least 5-fold in one mock subgenome compared to the other mock subgenome. The 12-mers that satisfied these criteria in fewer than ten out of 1,000 randomizations (*p* < 0.01) were considered significantly overrepresented.

Since we determined significance of overrepresentation for hundreds of 12-mers simultaneously, we needed to adjust for multiple testing. Using a false discovery rate approach [33], we verified that all 12-mers significantly overrepresented according to the permutation test had extremely low false discovery rates (*q* < 0.01). This can be explained by the high stringency of the overrepresentation criteria we set even before applying the permutation test. Thus, our dataset has few false positives after applying initial overrepresentation criteria (at least ten occurrences and at least 5-fold enrichment) and likely very few (if any) false positives after the permutation test.

After identifying significantly overrepresented 12-mers within each subgenome, we merged overlapping 12-mers to avoid scoring them twice. Using *sim4* with default parameters [34], we aligned all significantly overrepresented 12-mers identified for a subgenome against each other. The 12-mers with aligned regions of ≥8 bp (exact match) were merged to generate oligomers. This resulted in six groups of metamers, one for each subgenome (*E*_50_, *I*_50_, *E*_100_, *I*_100_, *E*_250_, and *I*_250_; Figure 1).

We next grouped all oligomers identified in each of the *E* subgenomes (*E*_50_, *E*_100_, and *E*_250_) and aligned them against each other using *sim4* with default parameters [34]. Again, oligomers with aligned regions of ≥8 bp (exact match) were merged. Oligomers identified in the three *I* subgenomes underwent similar treatment. This resulted in two groups of oligomers: *I-* and *E*-overrepresented oligomers (Figure 1).

Overrepresented oligomers were assigned to interspersed repetitive elements if both start and end genomic coordinates of oligomers were within interspersed repeats as annotated by Repeatmasker (RepBase Update 10.04, version 20050523). For overrepresented oligomers mapping to L1s, we also calculated their coordinates within L1 sequences. The 25 full-length consensus sequences of L1 families [35] were aligned using CLUSTALW [36] with default parameters to derive the L1 consensus sequence. The overrepresented oligomers were aligned to this consensus sequence using BLAST [37] with the following parameters: −F F, −W 7, −r 4, and −q −5.

### LDA.

To calculate the number of occurrences of a particular overrepresented oligomer in a subgenome, we counted the number of times at least one of the initial 12-mers used in “assembling” this oligomer was present in a subgenome. Several hits within an oligomer at a particular genomic location were counted only once. For instance, an overrepresented oligomer AAAAACAAGCAATG was created by merging two 12-mers, AAAAACAAGCAA and AAACAAGCAATG. If a subgenome had sequence AAAAACAAGCAATG at a particular genomic coordinate, it was counted only once, even though it had matches to two different initial 12-mers (AAAAACAAGCAA and AAACAAGCAATG). If a subgenome had sequence AAAAACAAGCAACC at some other genomic coordinate, it was also counted once because one 12-bp match (AAAAACAAGCAA) to the overrepresented oligomer could be found. If AAAAACAAGCAATG and AAAAACAAGCAACC were the only two occurrences of this overrepresented oligomer in a subgenome, its total count was 2 (this is just to illustrate how we counted overrepresented oligomers; in reality we required at least ten occurrences in a subgenome).

The counts of overrepresented oligomers in the ±50-kb, ±100-kb, and ±250-kb windows surrounding the TSSs were used to predict gene inactivation status. These counts formed a *p*-dimensional predictor vector *X* = *(X*_1_,...*X_p_)*, where *p* was equal to 110 + 138 = 248, the number of overrepresented oligomers for both the *E* and the *I* subgenome. Since the dimension exceeded the number of genes in the training set (Table 3), we first reduced the dimension by principal components analysis on the normalized predictor vector. Normalization consisted of subtracting the mean and dividing by the standard deviation for each predictor (vector coordinate). We used the first five principal components because they captured a substantial amount of the variability in the original data and were optimal in the subsequent classification analysis. Thus, features used in training and testing the LDA classifier formed a five-dimensional vector *Z* = (*Z*_1_,…*Z*_5_).

Following [38], the LDA direction *L* was computed using singular value decomposition of the matrix *W* ^−1/2^ *BW* ^−1/2^, where *W* and *B* are the within and between variance-covariance matrices of *Z,* respectively. The LDA score of a gene with features *Z*(*g*) is thus given by *λ*(*g*)=*L*′*Z*(*g*), and the gene is classified depending on the value of this score relative to a threshold *c*. The threshold is expressed by a convex combination of the average LDA scores for the two classes *(I* and *E)* in the training data,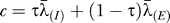 . The tuning parameter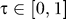 was selected to maximize the sum of correct classification rates for *E* genes and for *I* genes.

Correct classification rates on the training datasets were computed by leave-one-out cross-validation: at each round, one gene was withheld and the classifier was trained on the remaining genes, and then the withheld gene was classified. Correct classification rates for test sets were obtained by applying the trained classifier to the test sets.

## Supporting Information

Table S1 Gene and Contig Information for the Xp22 *E* and *I* Subgenomes (33 KB PDF)

Table S2 List of Overrepresented Oligomers (40 KB PDF)

Table S3 Gene Lists for Training and Test Datasets (63 KB PDF)

Table S4 Results of Classification When Only (GATA)_n_ Was Used (40 KB PDF)

### Accession Numbers

The GenBank (http://www.ncbi.nlm.nih.gov/Genbank) accession number for Hs.458197 is BE378480.

## Abbreviations

kb: kilobase
LDA: linear discriminant analysis
TSS: transcription start site
XCI: X chromosome inactivation
