# Supplementary material for: Genomic Environment Predicts Expression Patterns on the Human Inactive X Chromosome
Source: PLoS Genet. 2006 Sep 29;2(9):e151. doi: 10.1371/journal.pgen.0020151 (PMC1584270; doi:10.1371/journal.pgen.0020151)
Supplement: Table S1 — (33 KB PDF) [file pgen.0020151.st001.pdf]

Supplemental Table 1. Composition of Xp22 subgenomes

Percentage of sequence in repeats.

		I 50K	E 50K	I 100K	E 100K	I 250K	E 250K
**GC**		40.6	41.17	40.58	40.85	39.39	40.1
**SINEs**		15.85	15.79	12.19	13.14	10.49	10.8
	ALUs	13.25	14.04	9.44	11.44	7.62	9.4
	MIRs	2.6	1.74	2.75	1.69	2.87	1.43
**LINEs**		16.59	11.97	19.08	12.02	19.63	13.8
	LINE1	13.31	9.72	15.38	9.41	15.79	11.1
	LINE2	2.86	1.71	3.15	1.86	3.39	2.02
	L3/CR1	0.3	0.47	0.36	0.66	0.32	0.54
**LTR**		6.61	9.27	7.08	10.43	6.01	12.5
	MaLRs	3.88	4.25	4.04	4.21	4.04	5.62
	ERVL	1.31	1.17	1.48	1.6	1.14	1.48
	ERV_classI	1.33	3.74	1.34	4.42	0.57	5.27
	ERV_classII	0.1	0.1	0.2	0.2	0.22	0.11
**DNA**		4.39	3.93	4.61	3.76	4.97	3.58
	MER1_type	2.8	2.67	3.18	2.44	3.51	2.32
	MER2_type	0.97	0.78	0.8	0.9	0.75	0.9
**Simple repeats**		0.61	0.88	0.71	1.03	0.72	1.29
**Low complexity**		0.48	0.54	0.44	0.71	0.47	0.72

	Gene	Expression	TSS	Start of Coordinate	End of Coordinate	Contig number
**I 50K**	Hs.187608	1 / 9	10397719	10347719	10447719	1
	MID1	1 / 9	10398195	10348195	10448195	1
	HCCS	0 / 9	10889118	10839118	10939118	2
	H48827	1 / 9	11030019	10980019	11080019	3
	ARHGAP6	1 / 9	11443478	11393478	11493478	4

PRPS2	0 / 5	12569187	12519187	12619187	5
GLRA2	1 / 9	14307306	14257306	14357306	6
FANCB	0 / 9	14650848	14600848	14700848	7
MOSPD2	0 / 9	14651219	14601219	14701219	7
RAI2	0 / 9	17638484	17588484	17688484	8
Hs.220888	0 / 9	18124098	18074098	18174098	9
SCML2	0 / 9	18132458	18082458	18182458	9
Hs.149297	0 / 9	18863600	18813600	18913600	10
DKFZp686C0	0 / 9	18867606	18817606	18917606	10
PDHA1	0 / 6	19121707	19071707	19171707	11
CXorf23	1 / 9	19744081	19694081	19794081	12
RPS6KA3	0 / 9	20044407	19994407	20094407	13
YY2	0 / 9	21634259	21584259	21684259	14
SMS	0 / 6	21718524	21668524	21768524	14
FLJ37866	0 / 9	21950757	21900757	22000757	15
AA601738	1 / 9	23677260	23627260	23727260	16
PDK3	0 / 9	24243071	24193071	24293071	17
Hs.403937	0 / 9	24318005	24268005	24368005	17
POLA	0 / 9	24471720	24421720	24521720	18
Z39260	0 / 9	24583884	24533884	24633884	19

E 50K

NLGN4X	9 / 9	6005624	5955624	6055624	1
FLJ12417	9 / 9	6842305	6792305	6892305	2
HDHD1A	8 / 9	6925883	6875883	6975883	2
STS	9 / 9	6997232	6947232	7047232	2
Hs.186498	9 / 9	7123776	7073776	7173776	3
N74477	8 / 9	7188013	7138013	7238013	3
Hs.495638	9 / 9	7234805	7184805	7284805	3
PNPLA4	9 / 9	7705179	7655179	7755179	4
Hs.495641	9 / 9	8234774	8184774	8284774	5
MGC17403	9 / 9	13430899	13380899	13480899	6
RAB9A	9 / 9	13466997	13416997	13516997	6
SEDL	9 / 9	13512374	13462374	13562374	6
OFD1	6 / 6	13512536	13462536	13562536	6

Hs.41434	9 / 9	13556895	13506895	13606895	6
GPM6B	8 / 9	13716488	13666488	13766488	7
AA952971	9 / 9	13772392	13722392	13822392	7
FAM51A1	9 / 9	13807668	13757668	13857668	7
PIR	9 / 9	15271016	15221016	15321016	8
TMEM27	9 / 9	15442811	15392811	15492811	9
CA5BL	9 / 9	15480672	15430672	15530672	9
CA5B	9 / 9	15516081	15466081	15566081	9
Hs.121592	9 / 9	15614002	15564002	15664002	9
Hs.431654	9 / 9	15628315	15578315	15678315	9
AP1S2	9 / 9	15632757	15582757	15682757	9
Hs.431102	9 / 9	16370147	16320147	16420147	10
CALB3	9 / 9	16427937	16377937	16477937	10
CTPS2	9 / 9	16490700	16440700	16540700	10
SYAP1	9 / 9	16497411	16447411	16547411	10
CXORF15	9 / 9	16564216	16514216	16614216	10
RBBP7	9 / 9	16648094	16598094	16698094	10
ZFX	9 / 9	23929464	23879464	23979464	11

I 100K

Hs.187608	1 / 9	10397719	10297719	10497719	1
MID1	1 / 9	10398195	10298195	10498195	1
HCCS	0 / 9	10889118	10789118	10989118	2
H48827	1 / 9	11030019	10930019	11130019	2
GLRA2	1 / 9	14307306	14207306	14407306	3
FANCB	0 / 9	14650848	14550848	14750848	4
MOSPD2	0 / 9	14651219	14551219	14751219	4
RAI2	0 / 9	17638484	17538484	17738484	5
Hs.149297	0 / 9	18863600	18763600	18963600	6
DKFZp686C0	0 / 9	18867606	18767606	18967606	6
PDHA1	0 / 6	19121707	19021707	19221707	7
PDK3	0 / 9	24243071	24143071	24343071	8
Hs.403937	0 / 9	24318005	24218005	24418005	8

E 100K

NLGN4X	9 / 9	6005624	5905624	6105624	1

FLJ12417	9 / 9	6842305	6742305	6942305	2
HDHD1A	8 / 9	6925883	6825883	7025883	2
STS	9 / 9	6997232	6897232	7097232	2
PNPLA4	9 / 9	7705179	7605179	7805179	3
Hs.495641	9 / 9	8234774	8134774	8334774	4
MGC17403	9 / 9	13430899	13330899	13530899	5
RAB9A	9 / 9	13466997	13366997	13566997	5
SEDL	9 / 9	13512374	13412374	13612374	5
OFD1	6 / 6	13512536	13412536	13612536	5
TMEM27	9 / 9	15442811	15342811	15542811	6
CA5BL	9 / 9	15480672	15380672	15580672	6
CA5B	9 / 9	15516081	15416081	15616081	6
Hs.121592	9 / 9	15614002	15514002	15714002	6
Hs.431654	9 / 9	15628315	15528315	15728315	6
Hs.431102	9 / 9	16370147	16270147	16470147	7
CALB3	9 / 9	16427937	16327937	16527937	7

I 250K

HCCS	0 / 9	10889118	10639118	11139118	1
H48827	1 / 9	11030019	10780019	11280019	1
FANCB	0 / 9	14650848	14400848	14900848	2
MOSPD2	0 / 9	14651219	14401219	14901219	2
POLA	0 / 9	24471720	24221720	24721720	3
Z39260	0 / 9	24583884	24333884	24833884	3

E 250K

NLGN4X	9 / 9	6005624	5755624	6255624	1
FLJ12417	9 / 9	6842305	6592305	7092305	2
HDHD1A	8 / 9	6925883	6675883	7175883	2
STS	9 / 9	6997232	6747232	7247232	2
Hs.186498	9 / 9	7123776	6873776	7373776	2
N74477	8 / 9	7188013	6938013	7438013	2
Hs.121592	9 / 9	15614002	15364002	15864002	3
Hs.431654	9 / 9	15628315	15378315	15878315	3
AP1S2	9 / 9	15632757	15382757	15882757	3
