# Supplementary material for: Genomic Environment Predicts Expression Patterns on the Human Inactive X Chromosome
Source: PLoS Genet. 2006 Sep 29;2(9):e151. doi: 10.1371/journal.pgen.0020151 (PMC1584270; doi:10.1371/journal.pgen.0020151)
Supplement: Table S2 — (40 KB PDF) [file pgen.0020151.st002.pdf]

Supplemental Table 2. The list of overrepresented oligomers.

overrepresented oligomers	Repeat	%	labels	P value (autosomal test)
CAGGGATGCTGC	DNA/MER1_type	100	Escape	0
GAACATGTGGTATT	LINE/L1	90.91	Escape	0.001
TCATGTCTAAAA	LINE/L1	80	Escape	0.12
AAACAAGAAATG	LINE/L1	60	Escape	0.03
ACAGAAAATCAA	LINE/L1	60	Escape	0.008
AAGGGAGGGAGG	Low_complexity	60	Escape	0.023
CATCAGTTAAGG	LTR/ERV1	90	Escape	0.038
AAGCAAGATGGA	LTR/ERV1	80.95	Escape	0.002
AAATCAGTCTCC	LTR/ERV1	70	Escape	0
AGAACCCTCTCT	LTR/ERV1	70	Escape	0
TTTATTAAGAAA	LTR/ERV1	66.67	Escape	0.003
AAAGTGAAAGCA	LTR/ERV1	60	Escape	0
ATAATTTTGCAA	LTR/ERV1	60	Escape	0
CCTCCCATGACA	LTR/MaLR	100	Escape	0
ATCATGGGGGTG	LTR/MaLR	100	Escape	0.008
AATTGTAATCCCC	LTR/MaLR	100	Escape	0.01
GGGTATGTCTTTATTA	LTR/MaLR	98.67	Escape	0.001
TCTCATCTTGAA	LTR/MaLR	96.3	Escape	0.001
GAGCTACAATTCAAG	LTR/MaLR	93.75	Escape	0.001
CAGACTAATACA	LTR/MaLR	92.86	Escape	0.043
AAGACATACCTG	LTR/MaLR	90	Escape	0.04
CAGCATGAGAACA	LTR/MaLR	90	Escape	0
CTTTATAGCAGTGTGAAAATGGACTAAT	LTR/MaLR	86.82	Escape	0
CTGTGTCCTCAC	LTR/MaLR	84.62	Escape	0
TACAAGCCAAGGAG	LTR/MaLR	84.38	Escape	0
CAGGTCCCTCCCA	LTR/MaLR	82.61	Escape	0
CTCCCCAGCCAC	LTR/MaLR	80	Escape	0.001
AGAACTCACTCA	LTR/MaLR	80	Escape	0.032
GATAGATAGATAGATAGATA	Simple_repeat	94.09	Escape	0
ACATATATGTAT	Simple_repeat	78.95	Escape	0.118
CTAGGCCTCCCA	SINE/Alu	100	Escape	0.105
TCGGGAGTTCAAGA	SINE/Alu	100	Escape	0.096
AAGTGATTCTCG	SINE/Alu	100	Escape	0.029
GAGGCAGGGGAA	SINE/Alu	100	Escape	0.294
AGAGATGAGGTCT	SINE/Alu	100	Escape	0.158
CCGCCCGCCTCG	SINE/Alu	100	Escape	0.245
CAGCGCTTTGGG	SINE/Alu	100	Escape	0.028
ACACACCACCAC	SINE/Alu	100	Escape	0.044
CAGCTCACCGCAA	SINE/Alu	100	Escape	0.022
AGGTGGATCACG	SINE/Alu	100	Escape	0.196
CCACTATGCCTGG	SINE/Alu	100	Escape	0.034
ACCTCCCAGGCT	SINE/Alu	100	Escape	0.122
CAAGGCAGGAGG	SINE/Alu	100	Escape	0.135
GAGCTATGATTGCACCAC	SINE/Alu	100	Escape	0
GACTCAAGCAATCCACC	SINE/Alu	100	Escape	0
AGATTGCTTGAGGCCA	SINE/Alu	100	Escape	0.066
TACAGGTGCACACCACCA	SINE/Alu	100	Escape	0.041
AGGTGTGCACCAC	SINE/Alu	100	Escape	0.123
CAGCCTCCAACTCCTGG	SINE/Alu	100	Escape	0
CCAGGAGGTTGAGGCTGCA	SINE/Alu	99.38	Escape	0
AATTGCTTGAGCCTAGGAGTTCA	SINE/Alu	98.18	Escape	0.005
CCTCATGATCCGCCC	SINE/Alu	98	Escape	0.35
GGAGGCAGAGCTTGCAGT	SINE/Alu	97.3	Escape	0.49
GCAGCCTTGAACTCC	SINE/Alu	96.77	Escape	0.017
TGGTGGTGCACACCAC	SINE/Alu	96.43	Escape	0.118
AAACTCCTGGCC	SINE/Alu	96.15	Escape	0.031
AGTTTGAGGCTG	SINE/Alu	95	Escape	0.003
CAGGCTCAAGCA	SINE/Alu	93.33	Escape	0.04
AGCTCAAGCAAT	SINE/Alu	91.67	Escape	0.044
CCCCATCTCTAAAA	SINE/Alu	91.3	Escape	0.02
CCCAAGCAGCTGG	SINE/Alu	90.91	Escape	0.046
CATCACTGCACTC	SINE/Alu	87.88	Escape	0.096
CCAGCTACCTGGGA	SINE/Alu	86.96	Escape	0.034
GGATTACAGACATG	SINE/Alu	85.71	Escape	0.076
AAGAGGCTGAGGTGGAAGGAT	SINE/Alu	84.75	Escape	0

ACAGTGGTGTGA	SINE/Alu	84.62	Escape	0.002
AGAGACAAGGTCTCA	SINE/Alu	84.21	Escape	0
AGCTGGTCTTGA	SINE/Alu	81.82	Escape	0.009
CCCTGTTTCTAC	SINE/Alu	80	Escape	0.082
TCTCTATAAAAAA	SINE/Alu	78.57	Escape	0.019
CTACAAAAATAA	SINE/Alu	66.67	Escape	0
AAAATACACAAA	SINE/Alu	65.22	Escape	0.033
CAGTGGTTCTTCC			Escape	0
AAAGCCAGTTAC			Escape	0
AAACCATATCAC			Escape	0
GATTTTCAAAAA	DNA/MER1_type	20	Escape	0
ATTATTATTTTATTA	DNA/MER1_type	13.64	Escape	0.077
GTTATTTAAAAA	LINE/L1	40	Escape	0.005
AAAAGTTGAAAT	LINE/L1	40	Escape	0
ATAAATGAATAA	LINE/L1	36.36	Escape	0.053
CATTTATATAAAAATA	LINE/L1	28.12	Escape	0.001
ATATATTTTCAT	LINE/L1	21.43	Escape	0
ATAGGCCAGGCA	LINE/L2	10	Escape	0.008
CCTTCCTCCTCC	Low_complexity	30	Escape	0.023
AGGAAGGAGGGA	Low_complexity	30	Escape	0.097
ATAAAATTTTAA	Low_complexity	28.57	Escape	0.04
ATTTATATTTTA	Low_complexity	26.92	Escape	0.019
TATAAATAAAAATTAAA	Low_complexity	21.54	Escape	0.003
TTTAAATTAAAA	Low_complexity	20	Escape	0.221
ATATTAAAAAAA	Low_complexity	20	Escape	0.117
ATTTTTTAATTA	Low_complexity	20	Escape	0.043
AGAGAAAGAGAA	Low_complexity	20	Escape	0.383
AGGAGAAAGGAA	Low_complexity	10	Escape	0
TTTTAAATAAATGCAT	Low_complexity	7.69	Escape	0
AGATAAAGGAAA	LTR/ERV1	36.36	Escape	0
AATATTTGAAGA	LTR/ERV1	30	Escape	0.004
CCAAAAATAAAA	LTR/ERV1	18.18	Escape	0.031
AGAAGAAAATAA	LTR/ERV1	15.38	Escape	0.037
ATGGTGGTGATG	Simple_repeat	46.67	Escape	0.024
ATGTATATATAA	Simple_repeat	46.67	Escape	0.027
AGAGAGAGGGAGG	Simple_repeat	45.83	Escape	0.049
ACACACAGACACAC	Simple_repeat	41.03	Escape	0.047
TTTATATATAAA	Simple_repeat	40	Escape	0.011
AAAATAAATAATAA	SINE/Alu	24.44	Escape	0.101
TCTTAAAAAGAAAAGGAA	SINE/Alu	24.36	Escape	0
AAAAAGGAAAAG	SINE/Alu	20	Escape	0.176
TAATAAAAAATA	SINE/Alu	18.18	Escape	0.087
AAAACACACACA	SINE/Alu	12.5	Escape	0.067
AATATTAATAAT	SINE/Alu	10	Escape	0.011
TCAAAAGAAAAATAAT	SINE/Alu	9.38	Escape	0.096
GATGTTTAGCAGCAT	DNA/MER1_type	100	Inactivated	0
GCATTTCTAACAAG	DNA/MER1_type	98.46	Inactivated	0
GTGGTTCTCAAA	DNA/MER1_type	83.33	Inactivated	0.002
AGCAGCAGCAGC	DNA/MER1_type	56.25	Inactivated	0.107
TTACATAGGTATACA	LINE/L1	100	Inactivated	0.01
AGGGCATCCCTGTCTTGTGCC	LINE/L1	100	Inactivated	0.044
CAAGAGAAAGCA	LINE/L1	100	Inactivated	0.095
AATACCCTTTATTTC	LINE/L1	100	Inactivated	0.101
GTCTCAGCCCAA	LINE/L1	100	Inactivated	0.128
AAGAGCTCCTGAA	LINE/L1	100	Inactivated	0.032
GGCAACCTACAA	LINE/L1	100	Inactivated	0.046
ATGCAAAAATCCTC	LINE/L1	100	Inactivated	0.197
CATCTATTGAGAT	LINE/L1	100	Inactivated	0.465
GTTGGATTCCTA	LINE/L1	100	Inactivated	0.317
GACCAATGGAAC	LINE/L1	100	Inactivated	0.171
ATTTCATCCATG	LINE/L1	100	Inactivated	0.12
TCTAGTTCTAGATCCCTGAGG	LINE/L1	99.08	Inactivated	0.151
AGCACCATTTATTAAA	LINE/L1	97.22	Inactivated	0.04
CCATGCTCATGGA	LINE/L1	97.14	Inactivated	0.145
AAACCTAGGCAATACCATTCAGG	LINE/L1	96.77	Inactivated	0.057
CTCTGGCTGCCCTTAA	LINE/L1	96.67	Inactivated	0.059
TACAAGCCAGAAG	LINE/L1	96.43	Inactivated	0.014

CTTGACTCTTTATC	LINE/L1	96	Inactivated	0.008
GCAAACACATTCAAAAGCT	LINE/L1	95.89	Inactivated	0.009
TACAGTCCCACCAAC	LINE/L1	95.83	Inactivated	0.114
AGAAAATATTTGC	LINE/L1	95	Inactivated	0.116
AGGTTTGTCAAA	LINE/L1	95	Inactivated	0.097
GAAAACTGGCACAA	LINE/L1	94.44	Inactivated	0.06
AAAAGCTAGCAGAAGGCAAGAAATAAC	LINE/L1	94.44	Inactivated	0.017
ATATCCAGCCAAACTAAGCTTCATA	LINE/L1	94.39	Inactivated	0.017
CTTTCCATGTTTAGTGC	LINE/L1	93.94	Inactivated	0.011
GCATTAGGAGAAA	LINE/L1	93.75	Inactivated	0.017
ATGAACTCATCCTTT	LINE/L1	93.75	Inactivated	0.044
TAGAAAACCCCA	LINE/L1	93.33	Inactivated	0.206
TACATAATGGTAA	LINE/L1	92.86	Inactivated	0.013
AAAGGCCTTTGA	LINE/L1	92.86	Inactivated	0.021
GATCCCTTTACC	LINE/L1	92.31	Inactivated	0.014
ATGGGTCTTGAC	LINE/L1	92.31	Inactivated	0.005
TCCTGAAGGAAGC	LINE/L1	92.11	Inactivated	0.019
AAAAACTGGAAGCA	LINE/L1	92	Inactivated	0.196
CAAGCAAATGCTGAGAG	LINE/L1	91.89	Inactivated	0.024
AGGAAGAAACTG	LINE/L1	91.67	Inactivated	0.023
AAAATCTCTCAG	LINE/L1	91.67	Inactivated	0.028
CAAAGGGAAGCC	LINE/L1	91.67	Inactivated	0.02
CCCACTTATGAG	LINE/L1	90.91	Inactivated	0.02
ATGATCAAGTGG	LINE/L1	90.91	Inactivated	0.059
AACTCAGCTCTG	LINE/L1	90.91	Inactivated	0.087
CTATTCAACATAGTG	LINE/L1	90.91	Inactivated	0.079
CACACACCGGGG	LINE/L1	90.91	Inactivated	0.004
AATGGGCAAAAG	LINE/L1	90	Inactivated	0.038
AGTGTAAATTAG	LINE/L1	90	Inactivated	0.052
CAAAGAACATCTT	LINE/L1	90	Inactivated	0.078
GCTAACATCATA	LINE/L1	90	Inactivated	0.049
CTAATGTTGACA	LINE/L1	90	Inactivated	0.01
AATATCCCTGAT	LINE/L1	90	Inactivated	0.067
GCAAACTATCAC	LINE/L1	90	Inactivated	0.005
TTAGAAGGAAAA	LINE/L1	90	Inactivated	0.125
CTCTGGGACACA	LINE/L1	90	Inactivated	0.2
ACCTGAGAAAAA	LINE/L1	90	Inactivated	0.07
AAACTGCATCAAC	LINE/L1	88.46	Inactivated	0.003
GGAGAAATACCTA	LINE/L1	87.5	Inactivated	0.012
TCATCATCACTGG	LINE/L1	87.5	Inactivated	0.267
CATGCTAAAAAC	LINE/L1	86.36	Inactivated	0.075
AAGAATGTTGAATA	LINE/L1	86.05	Inactivated	0.011
TATACAAAAATCCTCA	LINE/L1	85.71	Inactivated	0.032
CTTTTCTGCATC	LINE/L1	85.71	Inactivated	0.123
TGCAGAGACACAC	LINE/L1	85.29	Inactivated	0.001
GAAAGGAACAAC	LINE/L1	85	Inactivated	0.021
TCATCCCTGGGA	LINE/L1	83.33	Inactivated	0.226
ACATGCACACAT	LINE/L1	81.82	Inactivated	0.07
CTTAAATGTAAA	LINE/L1	81.25	Inactivated	0.015
AAGAAAAGAATTTT	LINE/L1	80.43	Inactivated	0.002
AGGGACAATTTG	LINE/L1	80	Inactivated	0.128
CAGGCAGGAGAA	LINE/L1	80	Inactivated	0.051
ATGGAGCTGAAA	LINE/L1	80	Inactivated	0.138
CACCACCAGGCC	LINE/L1	80	Inactivated	0.015
AGCATGATGCCT	LINE/L1	80	Inactivated	0.009
AGTTTTTTCCAA	LINE/L1	80	Inactivated	0.368
ATTTTTTATTGC	LINE/L1	76.92	Inactivated	0.036
AAAGAAGGCCAT	LINE/L1	75	Inactivated	0.011
AATTTCTGTTCT	LINE/L1	72.73	Inactivated	0.019
TAACAATATTAA	LINE/L1	72.73	Inactivated	0.375
AATGAAGGAAAAA	LINE/L1	71.88	Inactivated	0.011
AAAAACAAGCAATG	LINE/L1	71.05	Inactivated	0.061
GAGAAAGAAATA	LINE/L1	68.75	Inactivated	0.05
AGACAAAAACCA	LINE/L1	66.67	Inactivated	0.033
CCATTTATTGAATA	LINE/L1	65	Inactivated	0.046
TGGAAAACAAAA	LINE/L1	64.29	Inactivated	0.011
AAAAACATGCCA	LINE/L1	60	Inactivated	0.003

CAGAAATAATAC	LINE/L1	60	Inactivated	0.005
AATGGAAAAACA	LINE/L1	50	Inactivated	0.052
AAAAAATCAACT	LINE/L1	50	Inactivated	0
GTGCCTGGCACA	LINE/L2	58.33	Inactivated	0.176
CCTAATCACCTC	LTR/MaLR	100	Inactivated	0
ATCCAGGATAAT	LTR/MaLR	100	Inactivated	0
GTGAGAACTCAC	LTR/MaLR	100	Inactivated	0.006
CAGGTTGGTCTC	SINE/Alu	100	Inactivated	0.076
GTGATCCGCCCACC	SINE/Alu	100	Inactivated	0.338
AAGGTCAGGAGT	SINE/Alu	100	Inactivated	0.402
GTACCACTGCAC	SINE/Alu	96.15	Inactivated	0.087
GAGATGGTGCCAC	SINE/Alu	95	Inactivated	0.047
AGCTCACGCCTG	SINE/Alu	92.31	Inactivated	0.04
CTGAGGTCAAGA	SINE/Alu	91.67	Inactivated	0.052
ACTTCACTCCAG	SINE/Alu	90	Inactivated	0.016
AGTTTGAGGCCA	SINE/Alu	90	Inactivated	0.002
TCTCCCGCCTCA	SINE/Alu	83.33	Inactivated	0.137
ACAGAGAGAGACT	SINE/Alu	76.67	Inactivated	0.026
CAAAAAAAAAGA	SINE/Alu	58.33	Inactivated	0.252
AAATGGGGATAA	SINE/MIR	100	Inactivated	0.045
CTCATTTAATCC	SINE/MIR	90	Inactivated	0.054
TCATCTGTAAAA	SINE/MIR	84.62	Inactivated	0.499
GGGCCGGGCGCA			Inactivated	0
AAAAATGTTTAA			Inactivated	0
AATTTTAAAAGG	DNA/MER2_type	10	Inactivated	0
AGAAATAGGAAT	LINE/L1	40	Inactivated	0.003
AGAAATAAAAGG	LINE/L1	40	Inactivated	0.011
TGATTTCTTTTTCA	LINE/L1	35.48	Inactivated	0.02
AAAACAAAGCAA	LINE/L1	33.33	Inactivated	0.011
AACATTTATTGATTT	LINE/L1	30.43	Inactivated	0.066
TAAATATATTCA	LINE/L1	30	Inactivated	0.001
AAGTATTTTATT	LINE/L1	30	Inactivated	0.123
CTCCCCACCCCC	LINE/L1	27.27	Inactivated	0.018
AAATTTAAAAAC	LINE/L1	20	Inactivated	0
AAAAAGATAAAA	LINE/L1	20	Inactivated	0.022
CAAAATATTTTC	LINE/L1	20	Inactivated	0.006
AGCTTTTTAAAA	LINE/L1	16	Inactivated	0
AAATAAAATGGA	LINE/L1	10	Inactivated	0.005
AAAAAAAAGTCC	LINE/L1	10	Inactivated	0.049
ATATTTATTAAG	LINE/L2	30	Inactivated	0.001
AGGAAGGAAAAG	Low_complexity	30	Inactivated	0.016
ATTTTCATTTTC	LTR/ERVL	9.09	Inactivated	0.006
AAAATGAAATTA	LTR/ERVL	9.09	Inactivated	0.032
AGAAGAAGAAGAA	Simple_repeat	20.48	Inactivated	0.121
AAAAATTCAAAA	SINE/Alu	38.89	Inactivated	0.008
AAAATTTAAAAG	SINE/Alu	30	Inactivated	0.058
ATTTTTTTGAAA	SINE/Alu	10	Inactivated	0.005
TTTAAAACAAAA	SINE/Alu	7.69	Inactivated	0.036
AAAATTTCAAAT	SINE/MIR	10	Inactivated	0.012
