# Supplementary material for: Genomic Environment Predicts Expression Patterns on the Human Inactive X Chromosome
Source: PLoS Genet. 2006 Sep 29;2(9):e151. doi: 10.1371/journal.pgen.0020151 (PMC1584270; doi:10.1371/journal.pgen.0020151)
Supplement: Table S3 — (63 KB PDF) [file pgen.0020151.st003.pdf]

Supplemental Table 3. The lists of genes in training and test data sets. Genes added to Xp22 training set are shown in blue.

50K

Training

NLGN4X	Escape
HDHD1A	Escape
FLJ12417	Escape
STS	Escape
Hs.186498	Escape
N74477	Escape
Hs.495638	Escape
PNPLA4	Escape
Hs.495641	Escape
MGC17403	Escape
RAB9A	Escape
SEDL	Escape
OFD1	Escape
GPM6B	Escape
Hs.41434	Escape
AA952971	Escape
FAM51A1	Escape
PIR	Escape
TMEM27	Escape
CA5BL	Escape
CA5B	Escape
AP1S2	Escape
Hs.121592	Escape
Hs.431654	Escape
CTPS2	Escape
Hs.431102	Escape
CALB3	Escape
SYAP1	Escape
CXORF15	Escape
RBBP7	Escape

100K

Training

Hs.187608	Inactivated
MID1	Inactivated
HCCS	Inactivated
H48827	Inactivated
GLRA2	Inactivated
FANCB	Inactivated
MOSPD2	Inactivated
RAI2	Inactivated
Hs.149297	Inactivated
DKFZp686C	Inactivated
PDHA1	Inactivated
PDK3	Inactivated
Hs.403937	Inactivated
NLGN4X	Escape
FLJ12417	Escape
HDHD1A	Escape
STS	Escape
PNPLA4	Escape
Hs.495641	Escape
MGC17403	Escape
RAB9A	Escape
SEDL	Escape
OFD1	Escape
TMEM27	Escape
CA5BL	Escape
CA5B	Escape
Hs.121592	Escape
Hs.431654	Escape
Hs.431102	Escape
CALB3	Escape

250K

Training

HCCS	Inactivated
H48827	Inactivated
FANCB	Inactivated
MOSPD2	Inactivated
POLA	Inactivated
Z39260	Inactivated
NLGN4X	Escape
HDHD1A	Escape
FLJ12417	Escape
STS	Escape
Hs.186498	Escape
N74477	Escape
AP1S2	Escape
Hs.121592	Escape
Hs.431654	Escape
PRRG1	Inactivated
TCTE1L	Inactivated
SRPX	Inactivated
TM4SF2	Inactivated
CHST7	Inactivated
EBP	Inactivated
OATL1	Inactivated
RBM3	Inactivated
SUV39H1	Inactivated
TIMM17B	Inactivated
OPHN1	Inactivated
DLG3	Inactivated
ZNF261	Inactivated
FSHPRH1	Inactivated
ARMCX3	Inactivated

ZFX	Escape
ARSD	Escape
USP9X	Escape
DUSP21	Escape
MID1	Inactivated
Hs.187608	Inactivated
HCCS	Inactivated
ARHGAP6	Inactivated
H48827	Inactivated
PRPS2	Inactivated
GLRA2	Inactivated
FANCB	Inactivated
MOSPD2	Inactivated
RAI2	Inactivated
SCML2	Inactivated
Hs.220888	Inactivated
Hs.149297	Inactivated
DKFZp686C0388	Inactivated
PDHA1	Inactivated
CXorf23	Inactivated
RPS6KA3	Inactivated
YY2	Inactivated
SMS	Inactivated
FLJ37866	Inactivated
AA601738	Inactivated
PDK3	Inactivated
Hs.403937	Inactivated
POLA	Inactivated
Z39260	Inactivated
PIGA	Inactivated
PRRG1	Inactivated
TCTE1L	Inactivated
SRPX	Inactivated
TM4SF2	Inactivated
GPR34	Inactivated
PRRG1	Inactivated
TCTE1L	Inactivated
SRPX	Inactivated
TM4SF2	Inactivated
GPR34	Inactivated
CHST7	Inactivated
PHF16	Inactivated
ZNF41	Inactivated
EBP	Inactivated
OATL1	Inactivated
RBM3	Inactivated
SUV39H1	Inactivated
TIMM17B	Inactivated
OPHN1	Inactivated
DLG3	Inactivated
ZNF261	Inactivated
Hs.374460	Inactivated
FSHPRH1	Inactivated
ARMCX3	Inactivated
AA663726	Inactivated
ZNF75	Inactivated
FHL1	Inactivated
HTATSF1	Inactivated
FMR1	Inactivated
IDS	Inactivated
CD99L2	Inactivated
CETN2	Inactivated
VBP1	Inactivated
TMLHE	Inactivated
ARSD	Escape
GPM6B	Escape
USP9X	Escape
DUSP21	Escape
**other Xp22**	
KIAA1280	Inactivated
AA663726	Inactivated
FHL1	Inactivated
HTATSF1	Inactivated
FMR1	Inactivated
IDS	Inactivated
CD99L2	Inactivated
CETN2	Inactivated
TMLHE	Inactivated
ARSD	Escape
SEDL	Escape
GPM6B	Escape
DUSP21	Escape
**other Xp22**	
KIAA1280	Inactivated
MID1	Inactivated
Hs.187608	Inactivated
ARHGAP6	Inactivated
M62076	Inactivated
SCML1	Inactivated
RAI2	Inactivated
SCML2	Inactivated
Hs.220888	Inactivated
Hs.149297	Inactivated
DKFZp686C	Inactivated
YY2	Inactivated
FLJ37866	Inactivated
XG	Escape
GYG2	Escape
ARSE	Escape
Hs.399941	Escape
Hs.495638	Escape
PNPLA4	Escape
Hs.495641	Escape
Hs.348675	Escape
MGC17403	Escape

CHST7	Inactivated
PHF16	Inactivated
ZNF41	Inactivated
EBP	Inactivated
OATL1	Inactivated
RBM3	Inactivated
SUV39H1	Inactivated
TIMM17B	Inactivated
OPHN1	Inactivated
DLG3	Inactivated
ZNF261	Inactivated
Hs.374460	Inactivated
FSHPRH1	Inactivated
ARMCX3	Inactivated
AA663726	Inactivated
ZNF75	Inactivated
FHL1	Inactivated
HTATSF1	Inactivated
FMR1	Inactivated
IDS	Inactivated
CD99L2	Inactivated
CETN2	Inactivated
IRAK1	Inactivated
MECP2	Inactivated
DNASE1L1	Inactivated
ATP6AP1	Inactivated
VBP1	Inactivated
TMLHE	Inactivated
**other Xp22**	
KIAA1280	Inactivated
M62076	Inactivated
EUROIMAGE	Inactivated
SCML1	Inactivated
PRDX4	Inactivated
SAT	Inactivated
ARHGAP6	Inactivated
M62076	Inactivated
SCML1	Inactivated
SCML2	Inactivated
Hs.220888	Inactivated
YY2	Inactivated
FLJ37866	Inactivated
PRDX4	Inactivated
SAT	Inactivated
PCYT1B	Inactivated
POLA	Inactivated
Z39260	Inactivated
XG	Escape
GYG2	Escape
ARSE	Escape
Hs.399941	Escape
Hs.186498	Escape
N74477	Escape
Hs.495638	Escape
Hs.348675	Escape
Hs.41434	Escape
AA952971	Escape
FAM51A1	Escape
AP1S2	Escape
CTPS2	Escape
SYAP1	Escape
CXORF15	Escape
RBBP7	Escape
**other X**	
GK	Inactivated
DMD	Inactivated
AA461044	Inactivated
TM4SF10	Inactivated
FLJ42925	Inactivated
SYTL5	Inactivated
RAB9A	Escape
Hs.41434	Escape
AA952971	Escape
CTPS2	Escape
Hs.431102	Escape
CALB3	Escape
SYAP1	Escape
CXORF15	Escape
RBBP7	Escape
**other X**	
GK	Inactivated
DMD	Inactivated
AA461044	Inactivated
TM4SF10	Inactivated
FLJ42925	Inactivated
SYTL5	Inactivated
Hs.61438	Inactivated
OTC	Inactivated
Hs.167510	Inactivated
MIG12	Inactivated
FLJ43479	Inactivated
FLJ20344	Inactivated
FLJ31752	Inactivated
ZC35F11	Inactivated
ZNF81	Inactivated
ZNF21	Inactivated
SLC38A5	Inactivated
FTSJ1	Inactivated
PORCN	Inactivated
Hs.432724	Inactivated
WDR13	Inactivated
GATA1	Inactivated
PCSK1N	Inactivated
SLC35A2	Inactivated
PIM2	Inactivated

PCYT1B	Inactivated
XG	Escape
GYG2	Escape
ARSE	Escape
Hs.399941	Escape
Hs.348675	Escape
**other X**	
GK	Inactivated
DMD	Inactivated
AA461044	Inactivated
TM4SF10	Inactivated
FLJ42925	Inactivated
SYTL5	Inactivated
Hs.61438	Inactivated
OTC	Inactivated
Hs.167510	Inactivated
MIG12	Inactivated
FLJ43479	Inactivated
FLJ22219	Inactivated
FLJ20344	Inactivated
FLJ31752	Inactivated
ZC35F11	Inactivated
SLC9A7	Inactivated
RP2	Inactivated
Hs.371977	Inactivated
RGN	Inactivated
SYN1	Inactivated
PFC	Inactivated
UXT	Inactivated
ZNF81	Inactivated
ZNF21	Inactivated
SLC38A5	Inactivated
FTSJ1	Inactivated
PORCN	Inactivated
Hs.432724	Inactivated
Hs.61438	Inactivated
OTC	Inactivated
Hs.167510	Inactivated
MIG12	Inactivated
FLJ43479	Inactivated
FLJ20344	Inactivated
FLJ31752	Inactivated
ZC35F11	Inactivated
SLC9A7	Inactivated
RP2	Inactivated
SYN1	Inactivated
PFC	Inactivated
UXT	Inactivated
ZNF81	Inactivated
ZNF21	Inactivated
SLC38A5	Inactivated
FTSJ1	Inactivated
PORCN	Inactivated
Hs.432724	Inactivated
WDR13	Inactivated
GATA1	Inactivated
PCSK1N	Inactivated
SLC35A2	Inactivated
PIM2	Inactivated
DKFZp761A	Inactivated
Hs.443531	Inactivated
KCND1	Inactivated
GRIPAP1	Inactivated
TFE3	Inactivated
JM11	Inactivated
JM4	Inactivated
WDRX1	Inactivated
Hs.496105	Inactivated
GPKOW	Inactivated
FLJ21687	Inactivated
DKFZp761A	Inactivated
Hs.443531	Inactivated
KCND1	Inactivated
GRIPAP1	Inactivated
TFE3	Inactivated
JM11	Inactivated
JM4	Inactivated
WDRX1	Inactivated
Hs.496105	Inactivated
GPKOW	Inactivated
FLJ21687	Inactivated
PLP2	Inactivated
CACNA1F	Inactivated
JM1	Inactivated
FOXP3	Inactivated
PPP1R3F	Inactivated
LOC158572	Inactivated
CLCN5	Inactivated
KIAA1202	Inactivated
NUDT10	Inactivated
LOC340602	Inactivated
NUDT11	Inactivated
GSPT2	Inactivated
PHF8	Inactivated
Hs.347445	Inactivated
FLJ42662	Inactivated
PRKWNK3	Inactivated
DT1P1A10	Inactivated
FGD1	Inactivated
FLJ10613	Inactivated
MAGED2	Inactivated
PFKFB1	Inactivated
Hs.13041	Inactivated
APEX2	Inactivated
ALAS2	Inactivated

WDR13	Inactivated	PLP2	Inactivated	LOC90736	Inactivated
GATA1	Inactivated	CACNA1F	Inactivated	MAGEH1	Inactivated
PCSK1N	Inactivated	JM1	Inactivated	Hs.40061	Inactivated
SLC35A2	Inactivated	FOXP3	Inactivated	W68846	Inactivated
PIM2	Inactivated	PPP1R3F	Inactivated	KLF8	Inactivated
DKFZp761A052	Inactivated	LOC158572	Inactivated	DKFZp686L	Inactivated
Hs.443531	Inactivated	CLCN5	Inactivated	SPIN3	Inactivated
KCND1	Inactivated	KIAA1202	Inactivated	SPIN2	Inactivated
GRIPAP1	Inactivated	NUDT10	Inactivated	ZXDB	Inactivated
TFE3	Inactivated	LOC340602	Inactivated	CXORF39	Inactivated
JM11	Inactivated	NUDT11	Inactivated	LOC92249	Inactivated
JM4	Inactivated	GSPT2	Inactivated	ASB12	Inactivated
WDRX1	Inactivated	UREB1	Inactivated	Hs.38448	Inactivated
Hs.496105	Inactivated	PHF8	Inactivated	MTMR8	Inactivated
GPKOW	Inactivated	Hs.347445	Inactivated	HCA127	Inactivated
FLJ21687	Inactivated	FLJ42662	Inactivated	YB62F01	Inactivated
PLP2	Inactivated	PRKWNK3	Inactivated	MGC21416	Inactivated
CACNA1F	Inactivated	DT1P1A10	Inactivated	STARD8	Inactivated
JM1	Inactivated	FGD1	Inactivated	EFNB1	Inactivated
FOXP3	Inactivated	FLJ10613	Inactivated	PJA1	Inactivated
PPP1R3F	Inactivated	MAGED2	Inactivated	ED1	Inactivated
LOC158572	Inactivated	PFKFB1	Inactivated	IGBP1	Inactivated
CLCN5	Inactivated	Hs.13041	Inactivated	Hs.442732	Inactivated
KIAA1202	Inactivated	APEX2	Inactivated	Hs.221457	Inactivated
NUDT10	Inactivated	ALAS2	Inactivated	ARR3	Inactivated
LOC340602	Inactivated	LOC90736	Inactivated	SNX12	Inactivated
NUDT11	Inactivated	MAGEH1	Inactivated	TNRC11	Inactivated
GSPT2	Inactivated	Hs.40061	Inactivated	NLGN3	Inactivated
SREB3	Inactivated	W68846	Inactivated	ITGB1BP2	Inactivated
HADH2	Inactivated	KLF8	Inactivated	OGT	Inactivated
UREB1	Inactivated	DKFZp686L	Inactivated	ACRC	Inactivated
PHF8	Inactivated	SPIN3	Inactivated	CXCR3	Inactivated
Hs.347445	Inactivated	SPIN2	Inactivated	NAP1L2	Inactivated
FLJ42662	Inactivated	ZXDB	Inactivated	RNF12	Inactivated
PRKWNK3	Inactivated	CXORF39	Inactivated	KIAA2022	Inactivated

DT1P1A10	Inactivated	LOC92249	Inactivated	Hs.353890	Inactivated
FGD1	Inactivated	ASB12	Inactivated	CXorf26	Inactivated
FLJ10613	Inactivated	Hs.38448	Inactivated	DKFZp564K	Inactivated
MAGED2	Inactivated	MTMR8	Inactivated	PGK1	Inactivated
PFKFB1	Inactivated	HCA127	Inactivated	TAF9L	Inactivated
Hs.13041	Inactivated	YB62F01	Inactivated	GPR23	Inactivated
APEX2	Inactivated	MGC21416	Inactivated	FLJ13042	Inactivated
ALAS2	Inactivated	STARD8	Inactivated	RPS6KA6	Inactivated
LOC90736	Inactivated	EFNB1	Inactivated	UNQ8193	Inactivated
MAGEH1	Inactivated	PJA1	Inactivated	SATL1	Inactivated
Hs.40061	Inactivated	ED1	Inactivated	TM4SF6	Inactivated
W68846	Inactivated	IGBP1	Inactivated	CSTF2	Inactivated
KLF8	Inactivated	Hs.442732	Inactivated	Hs.193951	Inactivated
DKFZp686L07201	Inactivated	Hs.221457	Inactivated	FLJ12687	Inactivated
SPIN3	Inactivated	ARR3	Inactivated	FLJ14084	Inactivated
SPIN2	Inactivated	SNX12	Inactivated	DRP2	Inactivated
ZXDB	Inactivated	TNRC11	Inactivated	Hs.381211	Inactivated
CXORF39	Inactivated	NLGN3	Inactivated	HNRPH2	Inactivated
LOC92249	Inactivated	ITGB1BP2	Inactivated	ARMCX1	Inactivated
ASB12	Inactivated	OGT	Inactivated	FLJ20811	Inactivated
Hs.38448	Inactivated	ACRC	Inactivated	YO64F11	Inactivated
MTMR8	Inactivated	CXCR3	Inactivated	Hs.303060	Inactivated
HCA127	Inactivated	PHKA1	Inactivated	ARMCX2	Inactivated
FLJ34366	Inactivated	NAP1L2	Inactivated	my048	Inactivated
FLJ12525	Inactivated	CHIC1	Inactivated	TMSNB	Inactivated
YB62F01	Inactivated	FLJ26979	Inactivated	FLJ12969	Inactivated
MGC21416	Inactivated	Hs.118526	Inactivated	GPRASP2	Inactivated
STARD8	Inactivated	DKFZp586J	Inactivated	Hs.496518	Inactivated
EFNB1	Inactivated	FLJ46440	Inactivated	Hs.53997	Inactivated
PJA1	Inactivated	RNF12	Inactivated	BEXL1	Inactivated
ED1	Inactivated	KIAA2022	Inactivated	MGC45400	Inactivated
IGBP1	Inactivated	Hs.353890	Inactivated	BEX2	Inactivated
Hs.442732	Inactivated	CXorf26	Inactivated	MGC23947	Inactivated
Hs.221457	Inactivated	DKFZp564K	Inactivated	NGFRAP1	Inactivated
ARR3	Inactivated	PGK1	Inactivated	TCEAL1	Inactivated

SNX12	Inactivated	TAF9L	Inactivated	PLP1	Inactivated
TNRC11	Inactivated	GPR23	Inactivated	RAB9B	Inactivated
NLGN3	Inactivated	FLJ13042	Inactivated	MGC39900	Inactivated
ITGB1BP2	Inactivated	RPS6KA6	Inactivated	Hs.110168	Inactivated
OGT	Inactivated	UNQ8193	Inactivated	DKFZp686C	Inactivated
ACRC	Inactivated	SATL1	Inactivated	FLJ36333	Inactivated
CXCR3	Inactivated	TM4SF6	Inactivated	LOC139231	Inactivated
RGAG4	Inactivated	CSTF2	Inactivated	FLJ33516	Inactivated
PHKA1	Inactivated	Hs.193951	Inactivated	FLJ10178	Inactivated
NAP1L2	Inactivated	FLJ12687	Inactivated	FLJ20298	Inactivated
CHIC1	Inactivated	FLJ14084	Inactivated	LOC92129	Inactivated
FLJ44396	Inactivated	DRP2	Inactivated	CLDN2	Inactivated
DKFZp686K1098	Inactivated	Hs.381211	Inactivated	ZCWCC2	Inactivated
FLJ26979	Inactivated	HNRPH2	Inactivated	AI650369	Inactivated
Hs.118526	Inactivated	ARMCX1	Inactivated	H66935	Inactivated
DKFZp586J1922	Inactivated	FLJ20811	Inactivated	PRPS1	Inactivated
FLJ46440	Inactivated	YO64F11	Inactivated	DSIPI	Inactivated
RNF12	Inactivated	Hs.303060	Inactivated	MID2	Inactivated
KIAA2022	Inactivated	ARMCX2	Inactivated	FLJ37392	Inactivated
Hs.353890	Inactivated	my048	Inactivated	FLJ41813	Inactivated
CXorf26	Inactivated	TMSNB	Inactivated	PSMD10	Inactivated
DKFZp564K142	Inactivated	FLJ12969	Inactivated	APG4A	Inactivated
PGK1	Inactivated	GPRASP2	Inactivated	KCNE1L	Inactivated
TAF9L	Inactivated	Hs.496518	Inactivated	CHRDL1	Inactivated
GPR23	Inactivated	Hs.53997	Inactivated	AMOT	Inactivated
FLJ13042	Inactivated	BEXL1	Inactivated	LRCH2	Inactivated
RPS6KA6	Inactivated	MGC45400	Inactivated	KLHL13	Inactivated
UNQ8193	Inactivated	BEX2	Inactivated	DKFZp686L	Inactivated
SATL1	Inactivated	MGC23947	Inactivated	Hs.186704	Inactivated
TM4SF6	Inactivated	NGFRAP1	Inactivated	IL13RA1	Inactivated
CSTF2	Inactivated	TCEAL1	Inactivated	RNF127	Inactivated
Hs.193951	Inactivated	PLP1	Inactivated	Hs.175048	Inactivated
FLJ12687	Inactivated	RAB9B	Inactivated	PGRMC1	Inactivated
FLJ14084	Inactivated	MGC39900	Inactivated	UBE2A	Inactivated
DRP2	Inactivated	Hs.110168	Inactivated	Hs.496662	Inactivated

Hs.381211	Inactivated	DKFZp686C	Inactivated	NRF	Inactivated
HNRPH2	Inactivated	FLJ36333	Inactivated	Hs.178536	Inactivated
ARMCX1	Inactivated	LOC139231	Inactivated	RPL39	Inactivated
FLJ20811	Inactivated	FLJ33516	Inactivated	ZNF183	Inactivated
YO64F11	Inactivated	FLJ10178	Inactivated	AKAP28	Inactivated
Hs.303060	Inactivated	FLJ20298	Inactivated	NKAP	Inactivated
ARMCX2	Inactivated	LOC92129	Inactivated	PEPP.2.	Inactivated
my048	Inactivated	CLDN2	Inactivated	ZBTB33	Inactivated
TMSNB	Inactivated	ZCWCC2	Inactivated	FLJ20716	Inactivated
FLJ12969	Inactivated	AI650369	Inactivated	LAMP2	Inactivated
GPRASP2	Inactivated	H66935	Inactivated	stSG42843	Inactivated
Hs.496518	Inactivated	PRPS1	Inactivated	C1GALT2	Inactivated
Hs.53997	Inactivated	DSIPI	Inactivated	THOC2	Inactivated
BEXL1	Inactivated	MID2	Inactivated	BIRC4	Inactivated
MGC45400	Inactivated	FLJ37392	Inactivated	STAG2	Inactivated
BEX2	Inactivated	FLJ41813	Inactivated	ODZ1	Inactivated
MGC23947	Inactivated	PSMD10	Inactivated	SMARCA1	Inactivated
NGFRAP1	Inactivated	APG4A	Inactivated	APLN	Inactivated
TCEAL1	Inactivated	KCNE1L	Inactivated	XPNPEP2	Inactivated
PLP1	Inactivated	CHRDL1	Inactivated	CXorf9	Inactivated
RAB9B	Inactivated	AMOT	Inactivated	ZDHHC9	Inactivated
MGC39900	Inactivated	LRCH2	Inactivated	FLJ11362	Inactivated
Hs.110168	Inactivated	KLHL13	Inactivated	ELF4	Inactivated
DKFZp686O1267	Inactivated	DKFZp686L	Inactivated	Hs.271940	Inactivated
FLJ36333	Inactivated	Hs.186704	Inactivated	Hs.424932	Inactivated
LOC139231	Inactivated	IL13RA1	Inactivated	SLC25A14	Inactivated
FLJ33516	Inactivated	RNF127	Inactivated	RBMX2	Inactivated
FLJ10178	Inactivated	Hs.175048	Inactivated	MST4	Inactivated
FLJ20298	Inactivated	PGRMC1	Inactivated	Hs.119889	Inactivated
LOC92129	Inactivated	UBE2A	Inactivated	FLJ38120	Inactivated
CLDN2	Inactivated	Hs.496662	Inactivated	Hs.291319	Inactivated
ZCWCC2	Inactivated	NRF	Inactivated	MBNL3	Inactivated
AI650369	Inactivated	Hs.178536	Inactivated	Hs.287979	Inactivated
H66935	Inactivated	RPL39	Inactivated	Hs.148401	Inactivated
PRPS1	Inactivated	ZNF183	Inactivated	Hs.268566	Inactivated

DSIPI	Inactivated	AKAP28	Inactivated	MGC16121	Inactivated
MID2	Inactivated	NKAP	Inactivated	W84737	Inactivated
FLJ37392	Inactivated	PEPP.2.	Inactivated	SLC9A6	Inactivated
FLJ41813	Inactivated	ZBTB33	Inactivated	Hs.205436	Inactivated
PSMD10	Inactivated	FLJ20716	Inactivated	FLJ12649	Inactivated
APG4A	Inactivated	LAMP2	Inactivated	ARHGEF6	Inactivated
KCNE1L	Inactivated	stSG42843	Inactivated	FLJ38034	Inactivated
CHRDL1	Inactivated	C1GALT2	Inactivated	RBMX	Inactivated
AMOT	Inactivated	THOC2	Inactivated	ATP11C	Inactivated
LRCH2	Inactivated	BIRC4	Inactivated	Hs.112784	Inactivated
KLHL13	Inactivated	STAG2	Inactivated	Hs.127679	Inactivated
DKFZp686L20145	Inactivated	ODZ1	Inactivated	Hs.31542	Inactivated
Hs.186704	Inactivated	SMARCA1	Inactivated	LDOC1	Inactivated
IL13RA1	Inactivated	APLN	Inactivated	Hs.6795	Inactivated
RNF127	Inactivated	XPNPEP2	Inactivated	CXORF40.	Inactivated
Hs.175048	Inactivated	CXorf9	Inactivated	U66043.	Inactivated
PGRMC1	Inactivated	ZDHHC9	Inactivated	FAM11A.	Inactivated
UBE2A	Inactivated	FLJ11362	Inactivated	U66046	Inactivated
Hs.496662	Inactivated	ELF4	Inactivated	N48339	Inactivated
NRF	Inactivated	Hs.271940	Inactivated	Hs.146087	Inactivated
Hs.178536	Inactivated	Hs.424932	Inactivated	MTM1	Inactivated
RPL39	Inactivated	SLC25A14	Inactivated	Hs.34079	Inactivated
ZNF183	Inactivated	RBMX2	Inactivated	MTMR1	Inactivated
AKAP28	Inactivated	MST4	Inactivated	M79236	Inactivated
NKAP	Inactivated	Hs.119889	Inactivated	Hs.99402	Inactivated
PEPP.2.	Inactivated	FLJ38120	Inactivated	Hs.124981	Inactivated
ZBTB33	Inactivated	Hs.291319	Inactivated	HMGB3	Inactivated
FLJ20716	Inactivated	MBNL3	Inactivated	LOC203547	Inactivated
LAMP2	Inactivated	Hs.287979	Inactivated	GABRE	Inactivated
stSG42843	Inactivated	Hs.148401	Inactivated	NSDHL	Inactivated
C1GALT2	Inactivated	Hs.268566	Inactivated	PNMA5	Inactivated
THOC2	Inactivated	MGC16121	Inactivated	PNMA6A	Inactivated
BIRC4	Inactivated	W84737	Inactivated	RAB39B	Inactivated
STAG2	Inactivated	LOC159091	Inactivated	F8A.	Inactivated
ODZ1	Inactivated	Hs.269127	Inactivated	FLJ25895.	Inactivated

SMARCA1	Inactivated
APLN	Inactivated
XPNPEP2	Inactivated
CXorf9	Inactivated
ZDHHC9	Inactivated
FLJ11362	Inactivated
ELF4	Inactivated
Hs.271940	Inactivated
Hs.424932	Inactivated
SLC25A14	Inactivated
RBMX2	Inactivated
MST4	Inactivated
Hs.119889	Inactivated
FLJ38120	Inactivated
Hs.291319	Inactivated
MBNL3	Inactivated
Hs.287979	Inactivated
Hs.148401	Inactivated
Hs.268566	Inactivated
MGC16121	Inactivated
W84737	Inactivated
LOC159091	Inactivated
Hs.269127	Inactivated
T91371	Inactivated
BC008642	Inactivated
FLJ23614	Inactivated
DDX26B	Inactivated
SLC9A6	Inactivated
Hs.205436	Inactivated
FLJ12649	Inactivated
ARHGEF6	Inactivated
FLJ38034	Inactivated
RBMX	Inactivated
ATP11C	Inactivated
Hs.112784	Inactivated
FLJ23614	Inactivated
DDX26B	Inactivated
SLC9A6	Inactivated
Hs.205436	Inactivated
FLJ12649	Inactivated
ARHGEF6	Inactivated
FLJ38034	Inactivated
RBMX	Inactivated
ATP11C	Inactivated
Hs.112784	Inactivated
Hs.127679	Inactivated
Hs.31542	Inactivated
LDOC1	Inactivated
Hs.6795	Inactivated
CXORF40.	Inactivated
U66043.	Inactivated
FAM11A.	Inactivated
U66046	Inactivated
N48339	Inactivated
Hs.146087	Inactivated
MTM1	Inactivated
Hs.34079	Inactivated
MTMR1	Inactivated
M79236	Inactivated
Hs.99402	Inactivated
Hs.124981	Inactivated
HMGB3	Inactivated
LOC203547	Inactivated
GABRE	Inactivated
NSDHL	Inactivated
PNMA5	Inactivated
PNMA6A	Inactivated
ZNF275	Inactivated
TREX2	Inactivated
ATP2B3	Inactivated
Hs.522189.	Inactivated
Hs.458197	Escape
MAOA	Escape
FUNDC1	Escape
Hs.232417	Escape
AA130835	Escape
SH3BGRL	Escape

Hs.127679	Inactivated
Hs.31542	Inactivated
LDOC1	Inactivated
Hs.6795	Inactivated
CXORF40.	Inactivated
U66043.	Inactivated
FAM11A.	Inactivated
U66046	Inactivated
N48339	Inactivated
Hs.146087	Inactivated
MTM1	Inactivated
Hs.34079	Inactivated
MTMR1	Inactivated
M79236	Inactivated
Hs.99402	Inactivated
Hs.124981	Inactivated
HMGB3	Inactivated
LOC203547	Inactivated
GABRE	Inactivated
NSDHL	Inactivated
PNMA5	Inactivated
PNMA6A	Inactivated
ZNF275	Inactivated
TREX2	Inactivated
ATP2B3	Inactivated
MGC29729	Inactivated
BC030106	Inactivated
DUSP9	Inactivated
TKTL1	Inactivated
FLNA	Inactivated
D60607	Inactivated
RPL10	Inactivated
TAZ	Inactivated
GDI	Inactivated
DXS9928E	Inactivated
TKTL1	Inactivated
FLNA	Inactivated
D60607	Inactivated
F8	Inactivated
HCBP6	Inactivated
Hs.6917	Inactivated
MTCP1	Inactivated
FLJ38567	Inactivated
H78350	Inactivated
RAB39B	Inactivated
F8A.	Inactivated
FLJ25895.	Inactivated
Hs.522189.	Inactivated
Hs.458197	Escape
Hs.282780	Escape
Hs.86849	Escape
Hs.229338	Escape
MAOA	Escape
FUNDC1	Escape
Hs.232417	Escape
AA130835	Escape
FLJ31610	Escape
SH3BGRL	Escape

F8	Inactivated
HCBP6	Inactivated
Hs.6917	Inactivated
MTCP1	Inactivated
FLJ38567	Inactivated
H78350	Inactivated
RAB39B	Inactivated
F8A.	Inactivated
FLJ25895.	Inactivated
Hs.522189.	Inactivated
Hs.458197	Escape
Hs.282780	Escape
Hs.86849	Escape
Hs.229338	Escape
DDX3X	Escape
MAOA	Escape
FUNDC1	Escape
Hs.232417	Escape
AA130835	Escape
KIAA0522	Escape
FLJ31610	Escape
T54860	Escape
BC014382	Escape
SH3BGRL	Escape
