# Supplementary material for: Genomic Environment Predicts Expression Patterns on the Human Inactive X Chromosome
Source: PLoS Genet. 2006 Sep 29;2(9):e151. doi: 10.1371/journal.pgen.0020151 (PMC1584270; doi:10.1371/journal.pgen.0020151)
Supplement: Table S4 — (40 KB PDF) [file pgen.0020151.st004.pdf]

Supplemental Table 4. Success rates of classification, when only $(GATA)_n$ was used.

	50 kb	100 kb	250 kb
Training set (largely Xp22)			
τ	0.95	0.93	0.26
Success in *E*	21%	24%	62%
Success in *I*	95%	86%	86%
Xp22 test set			
τ	0.29	0.03	0.05
Success in *E*	60%	81%	83%
Success in *I*	86%	85%	100%
Other X test set			
τ	0.07	0.17	0.15
Success in *E*	29%	50%	83%
Success in *I*	86%	65%	50%
Whole X training set			
τ	0.26	0.89	0.11
Success in *E*	33%	30%	70%
Success in *I*	77%	86%	69%
